# Supplementary material for: Spatiotemporal development of expanding bacterial colonies driven by emergent mechanical constraints and nutrient gradients
Source: Nat Commun. 2025 May 26;16:4878. doi: 10.1038/s41467-025-60004-z (PMC12106844; doi:10.1038/s41467-025-60004-z)
Supplement: Supplementary file 1 — Supplementary Information [file 41467_2025_60004_MOESM1_ESM.pdf]

Spatiotemporal development of expanding bacterial colonies driven by emergent mechanical constraints and nutrient gradients

Supplementary Information

Harish Kannan, Hui Sun, Mya Warren, Tolga Çağlar, Pantong Yao, Brian R. Taylor, Kinshuk Sahu, Daotong Ge, Matteo Mori, David Kleinfeld, JiaJia Dong, Bo Li, and Terence Hwa.

Contents

Supplementary Figures: Fig. S1-S15 2

Supplementary Notes 1-5 17

1 Supplementary Note 1: An Agent-Based Model for Cell Activities . . . 17
1.1 Cell growth, division, and movement . . . 17
1.2 Interaction forces . . . 17
2 Supplementary Note 2: A Continuum Model for Metabolism . . . 19
2.1 Metabolic model to determine cell growth rate . . . 19
2.2 Reaction-diffusion model for spatiotemporal dynamics of nutrients in (1+1)-dimensions . . 19
2.3 Model for cell maintenance . . . 22
2.4 Model for tracking nutrient starvation and predicting cell death within colony . . . 23
3 Supplementary Note 3: Numerical Methods and Computer Implementation . . . 25
3.1 A (1+1)-dimensional approximation . . . 25
3.2 Overall time iteration . . . 25
3.3 Numerical solution to reaction-diffusion equations . . . 25
4 Supplementary Note 4: Effects of (1+1)-dimensional geometry . . . 27
5 Supplementary Note 5: The effect of buffer concentration on toxicity due to acetate excretion . . . 31

Supplementary Tables 1-3 33

Supplementary References 35

Supplementary Figures

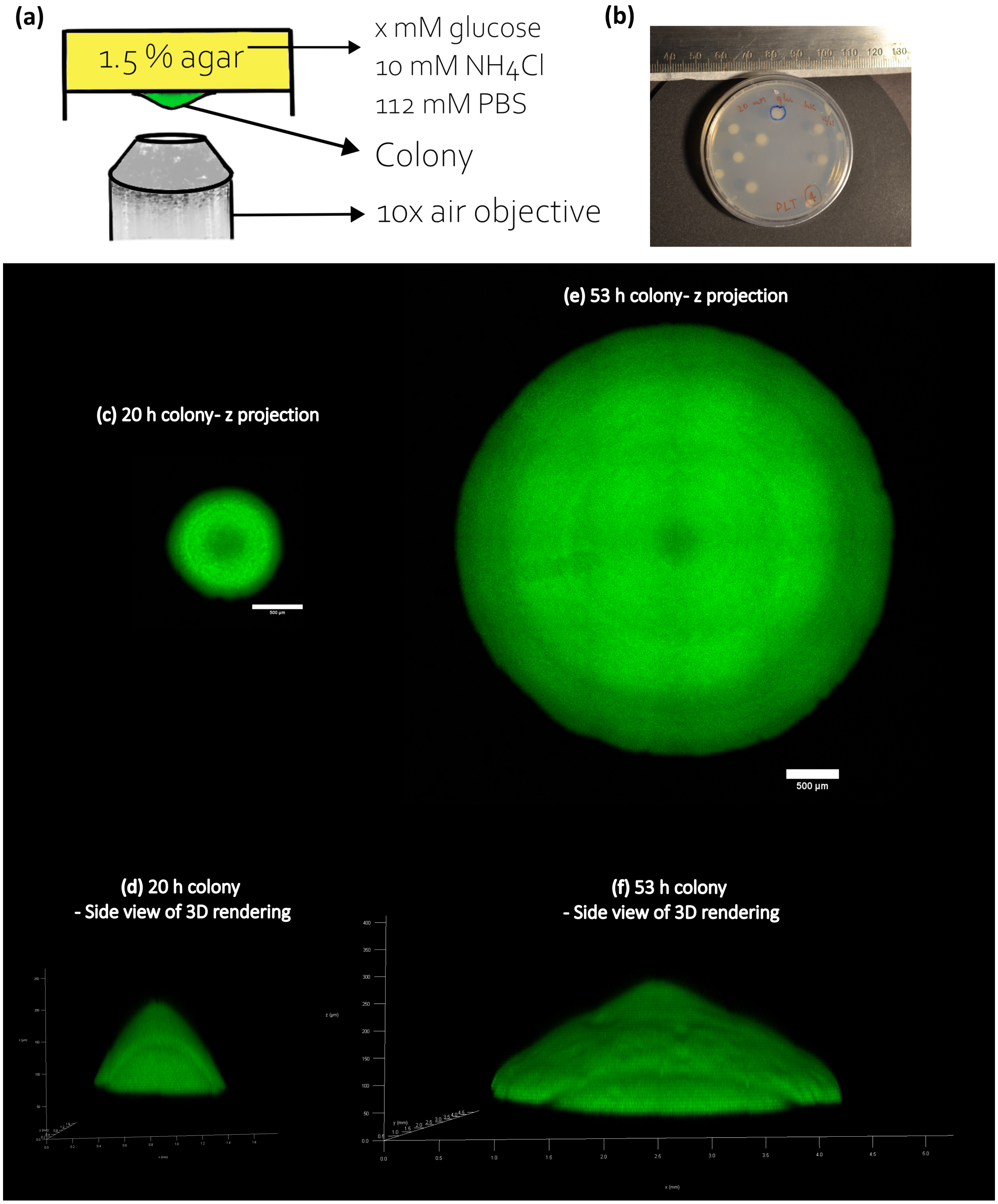

Fig. S1: ***Confocal microscopy images of E. coli colonies.*** **(a)** An illustration of the experimental set up to measure the dimensions of a colony growing on an agar plate using an inverted confocal microscope. **(b)** A photograph of a typical 60 mm x 15 mm agar plate in our experiments with E. coli colonies at $\sim$ 2 days post-inoculation. The number of colonies per plate is kept low at roughly $\sim$ 10 colony forming units (c.f.u.). **(c)** z-projection and **(d)** side-view of 3D rendering from microscopy images of a $\sim$ 20 h old EQ59 E. coli colony (see Supplementary Movie 1). **(e)** z-projection and **(f)** side-view of 3D rendering from microscopy images of a $\sim$ 53 h old EQ59 colony (see Supplementary Movie 1). 1.5 % (w/v) agar plates prepared with 20 mM glucose, 10 mM ammonium chloride, and 112 mM phosphate buffer were used.

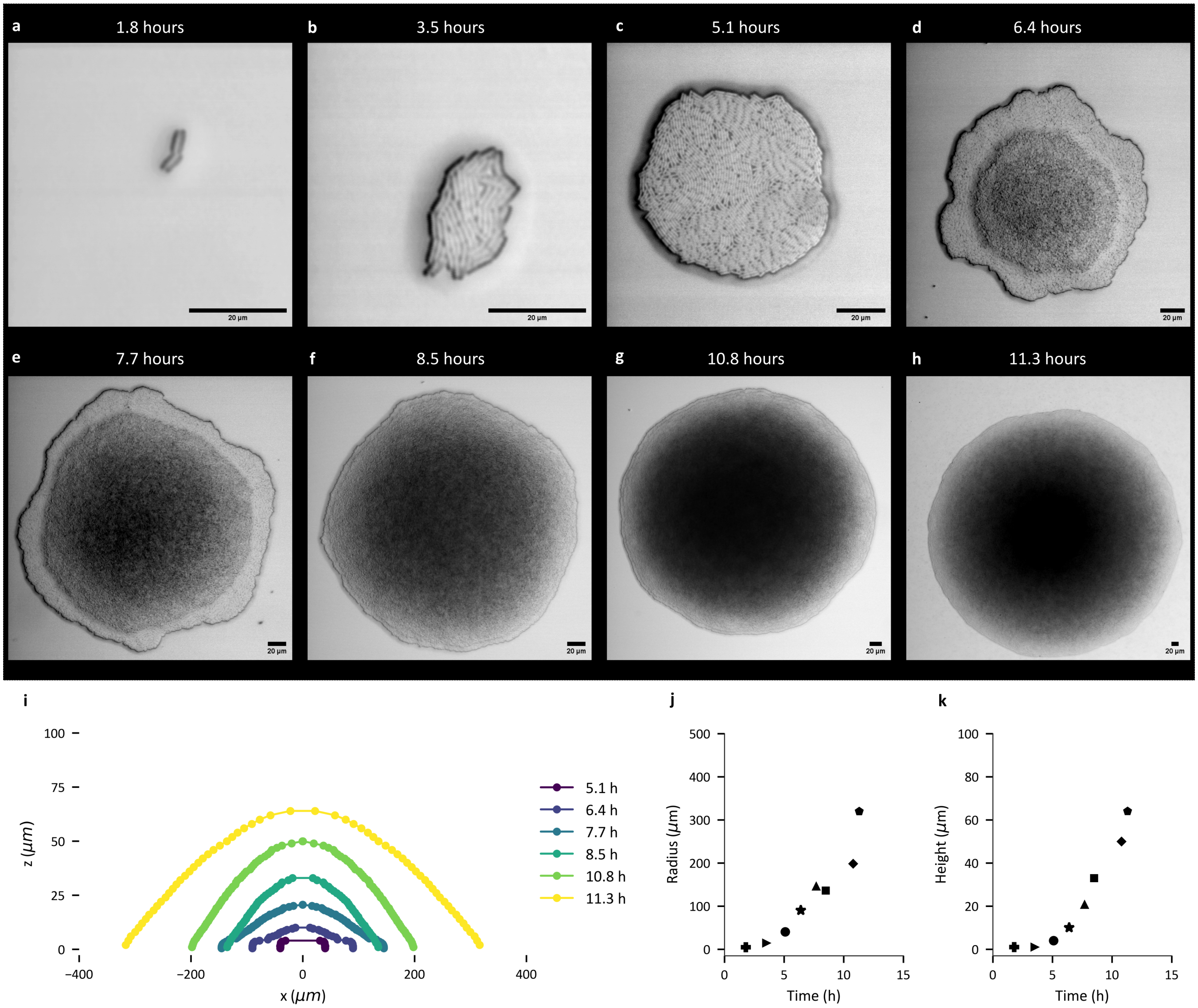

Fig. S2: ***Dynamics of early colony expansion.*** **(a-h)** Brightfield images of EQ59 *E. coli* colonies at various times during the first 12 hours post-inoculation on a 1.5 % agar minimal media glucose plate with 20 mM glucose. **(i)** Cross-sectional profiles, **(j)** radius and **(k)** height of young colonies. Each colony in (a-i) and each data point in (j,k) are different biological replicates.

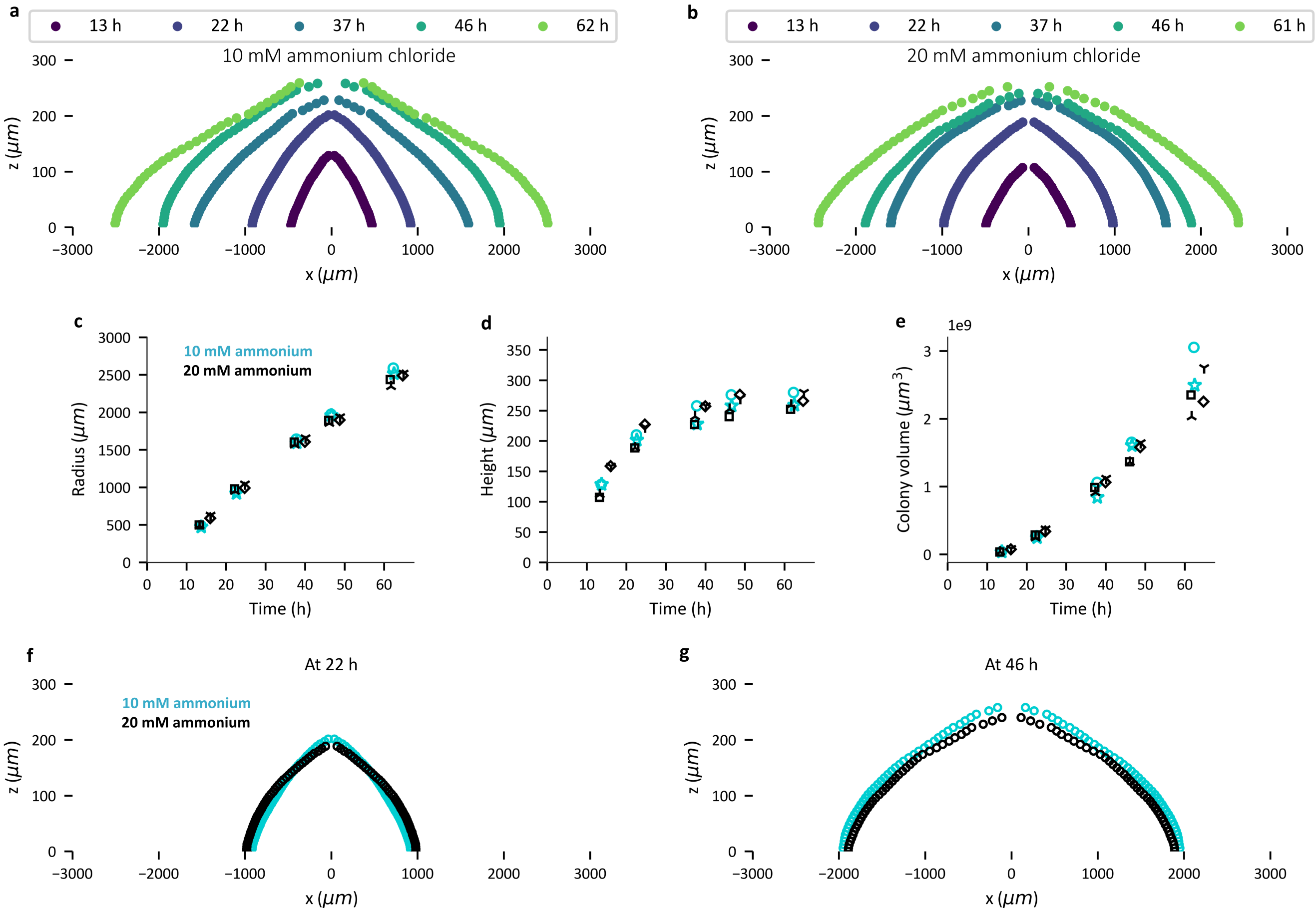

Fig. S3: *Colony expansion is not limited by the initial concentration of nitrogen source.* Expansion dynamics of EQ59 *E. coli* colonies on 1.5 % (w/v) agar plates prepared with 20 mM glucose, a defined ammonium chloride concentration (10 mM, 20 mM), and 112 mM phosphate buffer at various times post-inoculation as a single cell. The cross-sectional profile of a colony grown on a minimal media hard agar plate with 10 mM ammonium chloride **(a)** and 20 mM ammonium chloride **(b)** as nitrogen source shown for various times (coded by color) post-inoculation. The radius (μm) **(c)**, height (μm) **(d)**, and volume (μm^3) **(e)** of the colonies plotted against the time (h) post-inoculation. Cyan symbols represent colonies grown on minimal media plates with 10 mM ammonium chloride while black symbols represent colonies with 20 mM ammonium chloride. Each shape of symbol represents an individual biological replicate. The cross-sectional profile of a colony grown with 10 mM ammonium (cyan) and 20 mM ammonium (black) at $\sim$22 h **(f)** and $\sim$46h post-inoculation **(g)**.

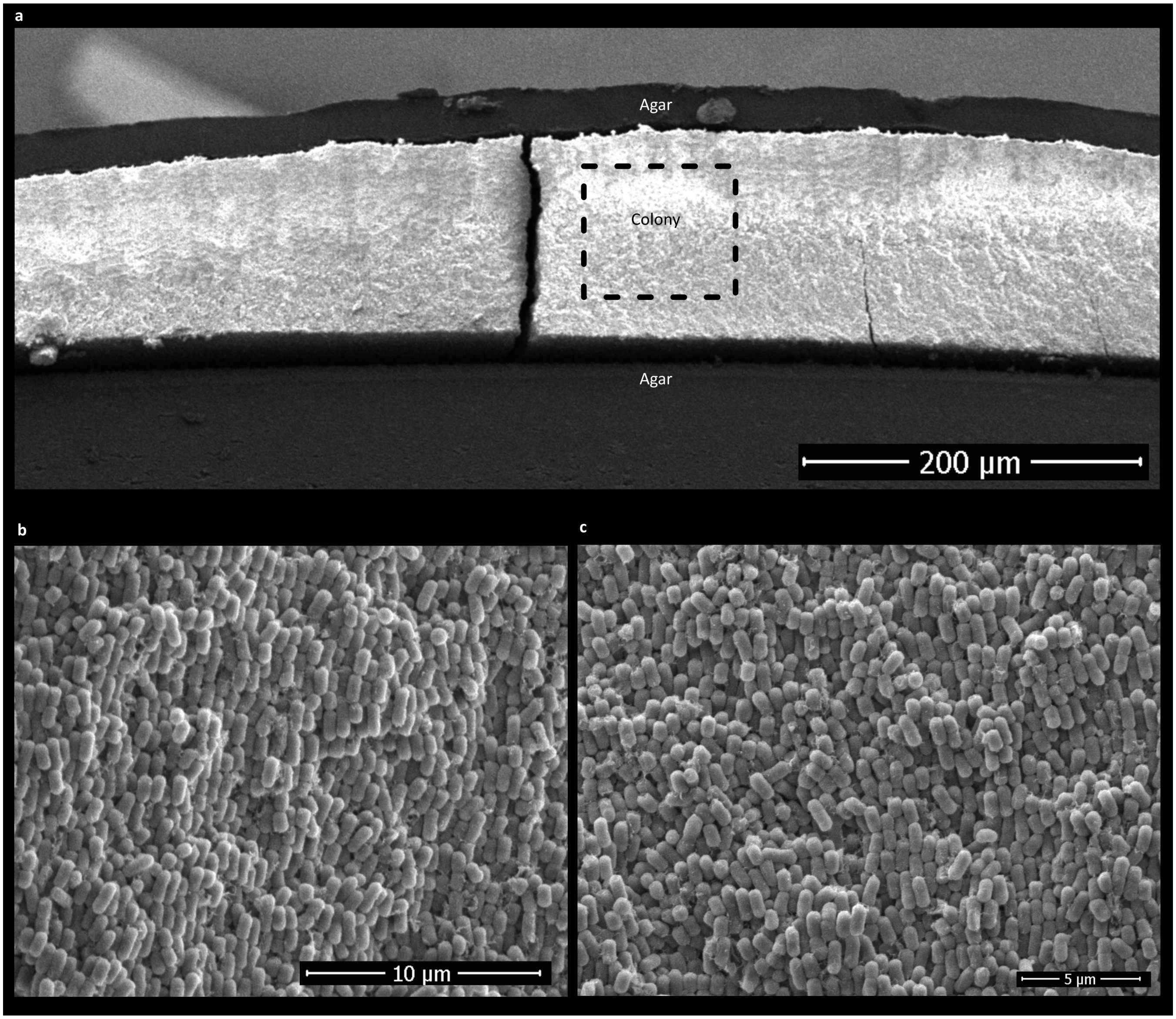

Fig. S4: ***Vertical orientation of cells in colony interior.*** **(a)** Macroscopic and **(b,c)** zoomed-in views of two different spatial locations in the colony cross-section of a $\sim$2 day old colony grown in 20 mM glucose minimal media agar obtained using scanning electron microscopy (see Methods in main text). Due to the lack of a EPS matrix in our colonies, the colony tends to structurally fragile during sample preparation for SEM and sometimes develops cracks (as seen by the black vertical dark line in panel a).

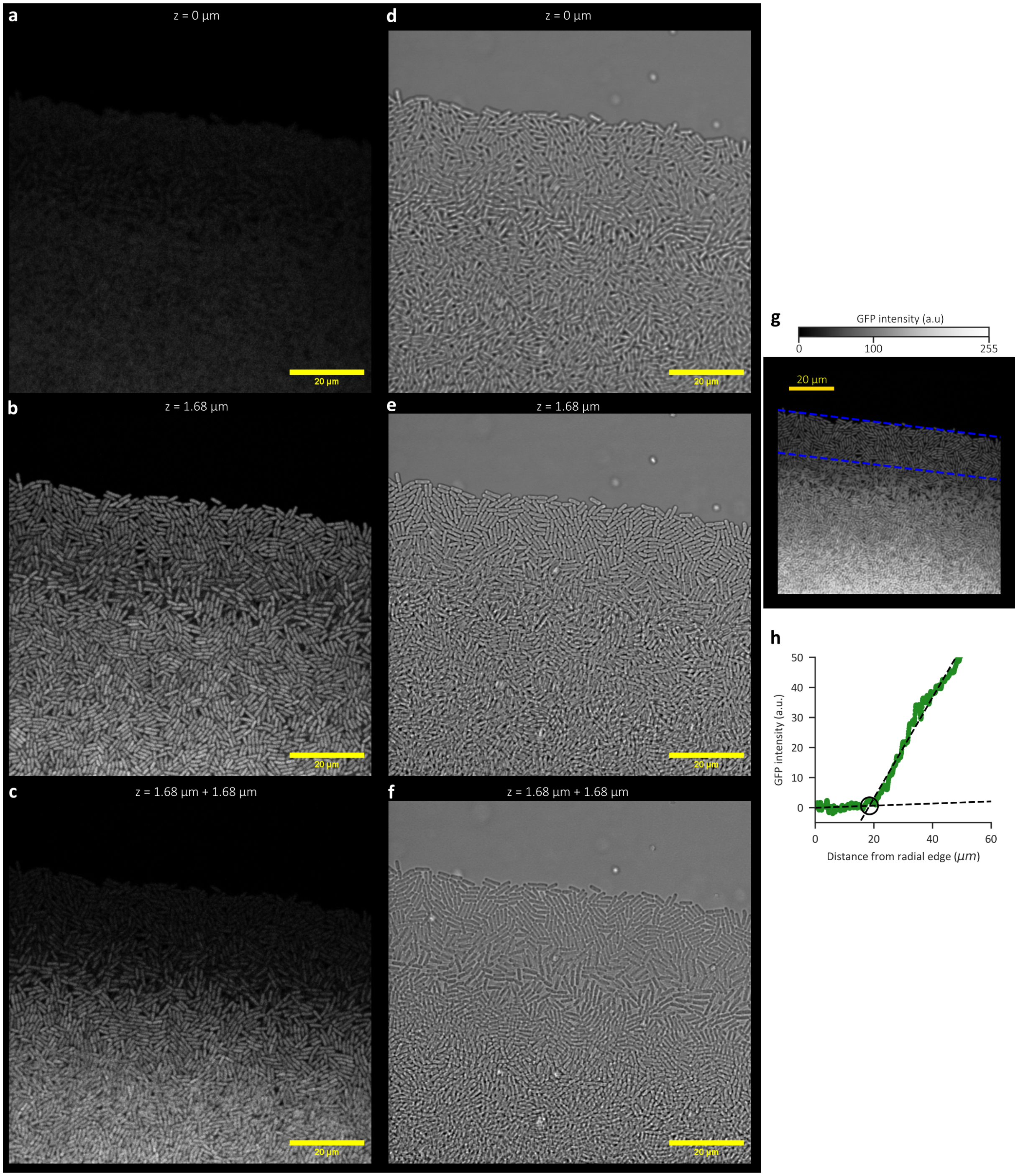

Fig. S5: ***Horizontally oriented monolayer of cells at radial periphery of colony.*** **(a-c)** GFP fluorescence at the radial edge of a $\sim$2-day old colony (grown with 20 mM glucose) obtained at high magnification using a confocal microscope (see Methods) at three different z positions and the corresponding brightfield images are shown in **(d-f)**. Intermediate z positions for the corresponding images are shown in Supplementary Movies 3,4 and it is observed that the fluorescence in the 20 µm peripheral region goes out of focus within $\sim$1-2 µm of z-dimension thickness indicating that is a single layer of cells. **(g)** GFP fluorescence intensity summed over the entire z-stack i.e., a summed z-projection of GFP fluorescence near the radial edge of a $\sim$2 day old colony grown with 20 mM glucose. Blue dashed lines mark the boundaries of the monolayer region identified using the spatial profile of GFP intensity where there is a sharp change in the GFP intensity profile (see panel h). **(h)** The GFP intensity profile (in green) as a function of the distance from the radial edge for the summed z-projection shown in panel g. First, the boundary of the colony is determined using a straight-line fit (see panel g) and then to obtain the GFP intensity at specified distance d from radial edge, the GFP intensity is averaged along the parallel straight line at a distance d from the line determining the colony boundary. The two black dashed lines represents a straight line fit of the GFP intensity between [0, x µm] and [x µm, 60 µm] correspondingly, where x here chosen as 18 µm and the intersection point of these two lines is used to estimate the monolayer width.

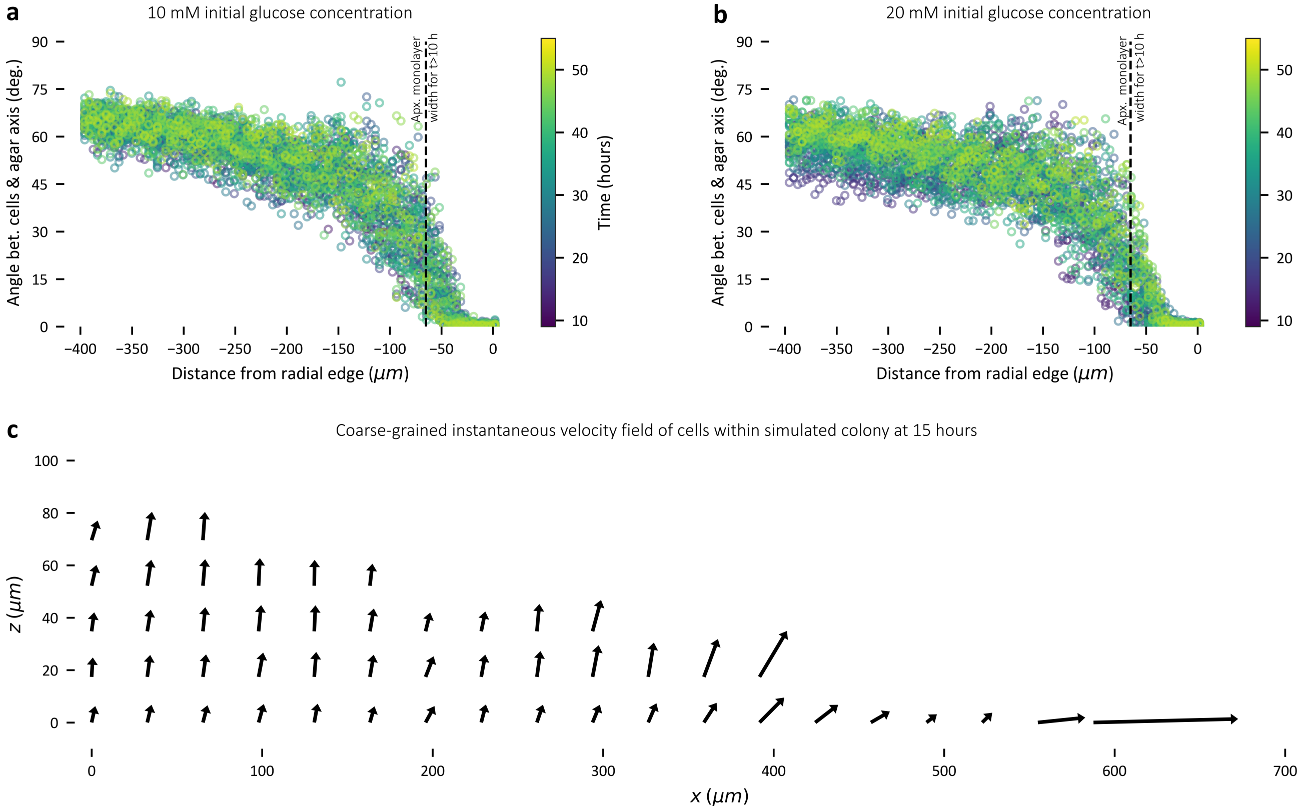

Fig. S6: ***Orientation of cells within simulated colony.*** Angle made by cells with the horizontal axis (agar axis) plotted as a function of their distance from peripheral edge for **(a)** 10 mM initial glucose concentration and **(b)** 20 mM initial glucose concentration simulations. The angle plotted is averaged over the vertical sections of the colony at a particular distance from either peripheral edge of the colony. Zero degrees represent horizontal orientation, and 90 degrees represent vertical orientation. A distance of zero represents the peripheral edge and negative values indicates moving into the colony interior. **(c)** Snapshot of coarse-grained velocity field of cells within a 15 h old (1+1)-dimensional simulated colony with 10 mM initial glucose concentration.

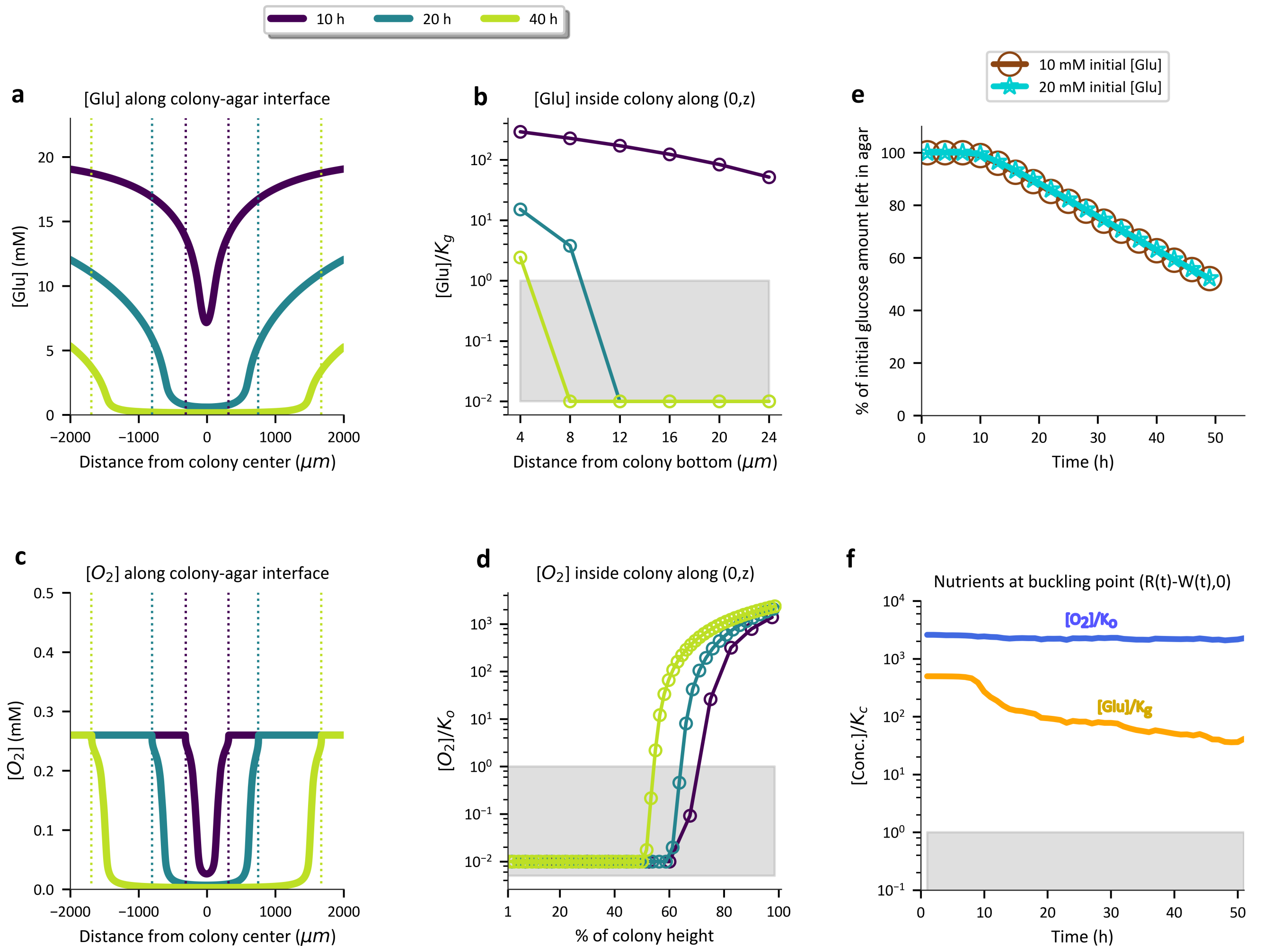

Fig. S7: ***Spatiotemporal dynamics of glucose and oxygen along the agar surface and inside simulated colony.*** **(a)** The glucose concentration (mM) along the colony-agar interface plotted against the distance (µm) from colony center at 10 h, 20 h and 40 h stages of colony development (time coded by color). The colored vertical dotted lines indicate the colony edge at the corresponding stages of colony development. **(b)** Glucose concentration normalized by K_g along the central vertical axis of the colony plotted against the distance (µm) from colony bottom at 10 h, 20 h and 40 h stages of colony development (time coded by color). The grey shaded region represents concentrations below the Monod constant K_g. **(c)** The oxygen concentration (mM) along the colony-agar interface plotted against the distance (µm) from colony center at 10 h, 20 h and 40 h stages of colony development (time coded by color). The colored vertical dotted lines indicate the colony edge at the corresponding stages of colony development. **(d)** The oxygen concentration normalized by K_o along the central vertical axis of the colony plotted against the distance from colony bottom expressed as percentage of colony height at 10 h, 20 h and 40 h stages of colony development (time coded by color). The grey shaded region represents concentrations below the Monod constant K_o. Results shown in panels a-d are for simulations with 20 mM initial glucose concentration. **(e)** The percentage of the initial amount of glucose (glucose concentration integrated over agar area) left in agar plotted as a function of time for simulations with 10 mM and 20 mM initial glucose concentration in agar. **(f)** The concentration of glucose (yellow) and oxygen (blue) at the buckling point on the colony-agar interface i.e., the spot where the colony is no longer a monolayer is plotted against time of colony development (h). Note that the buckling point is given by R(t)-W(t), where R(t) and W(t) represent the radius and the monolayer width at time t respectively). The grey shaded region represents nutrient concentrations below the corresponding Monod constant. Results shown in panel f are for simulations with 10 mM initial glucose concentration.

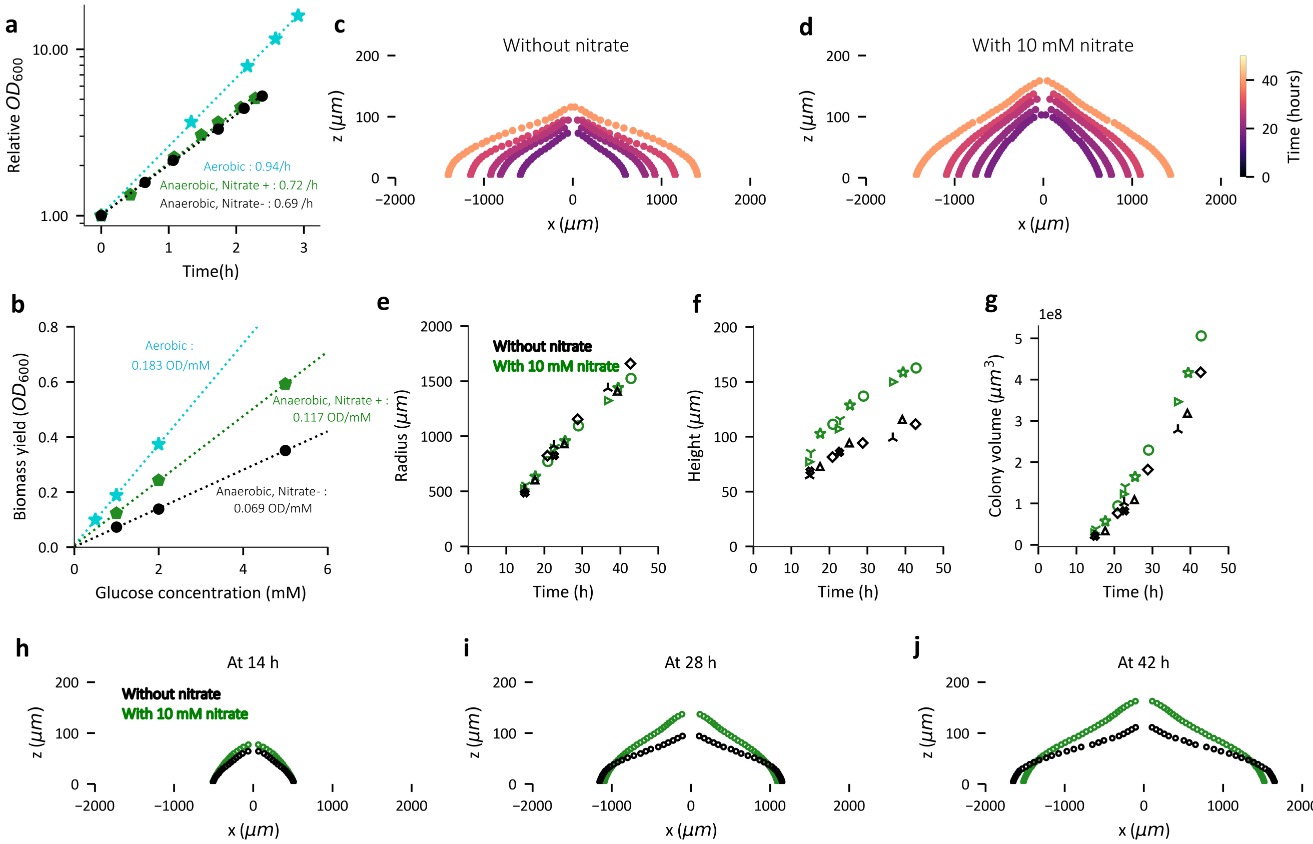

Fig. S8: ***Nitrate supplementation improves vertical expansion of colony.*** Batch culture growth **(a)**, and glucose-biomass conversion **(b)** i.e., biomass yield (OD_{600}/mM of glucose) for wild-type NCM3722 cells (parent strain of EQ59) grown in glucose minimal medium aerobically (cyan), anaerobically with 10 mM sodium nitrate supplementation (green) and anaerobically without nitrate supplementation (black). In panel a, the growth rate estimated from exponential fit (dotted lines) to data (symbols) is indicated in the legend for the corresponding culture conditions. In panel b, the biomass yield in units of OD_{600}/mM of glucose estimated from linear fit (dotted lines) to data (symbols) is indicated in the legend for the corresponding culture conditions. Expansion dynamics of EQ59 *E. coli* colonies on 1.5 % (w/v) agar plates prepared with 5 mM glucose minimal media with 10 mM sodium nitrate at various times post-inoculation as a single cell. The cross-sectional profile of colonies grown on a minimal media hard agar plate without nitrate **(c)** and with 10 mM nitrate **(d)** shown for various times (coded by color) post-inoculation. The radius (µm) **(e)**, height (µm) **(f)**, and volume (µm^3) **(g)** of the colonies with (green symbols) and without nitrate (black symbols) plotted against the time (h) post-inoculation. For (e-g) each type of symbol represents an individual biological replicate. **(h-j)** Comparison of cross-section profile of colonies grown without nitrate and with nitrate at $\sim$14 h **(h)**, $\sim$28 h **(i)**, and $\sim$42 h **(j)**.

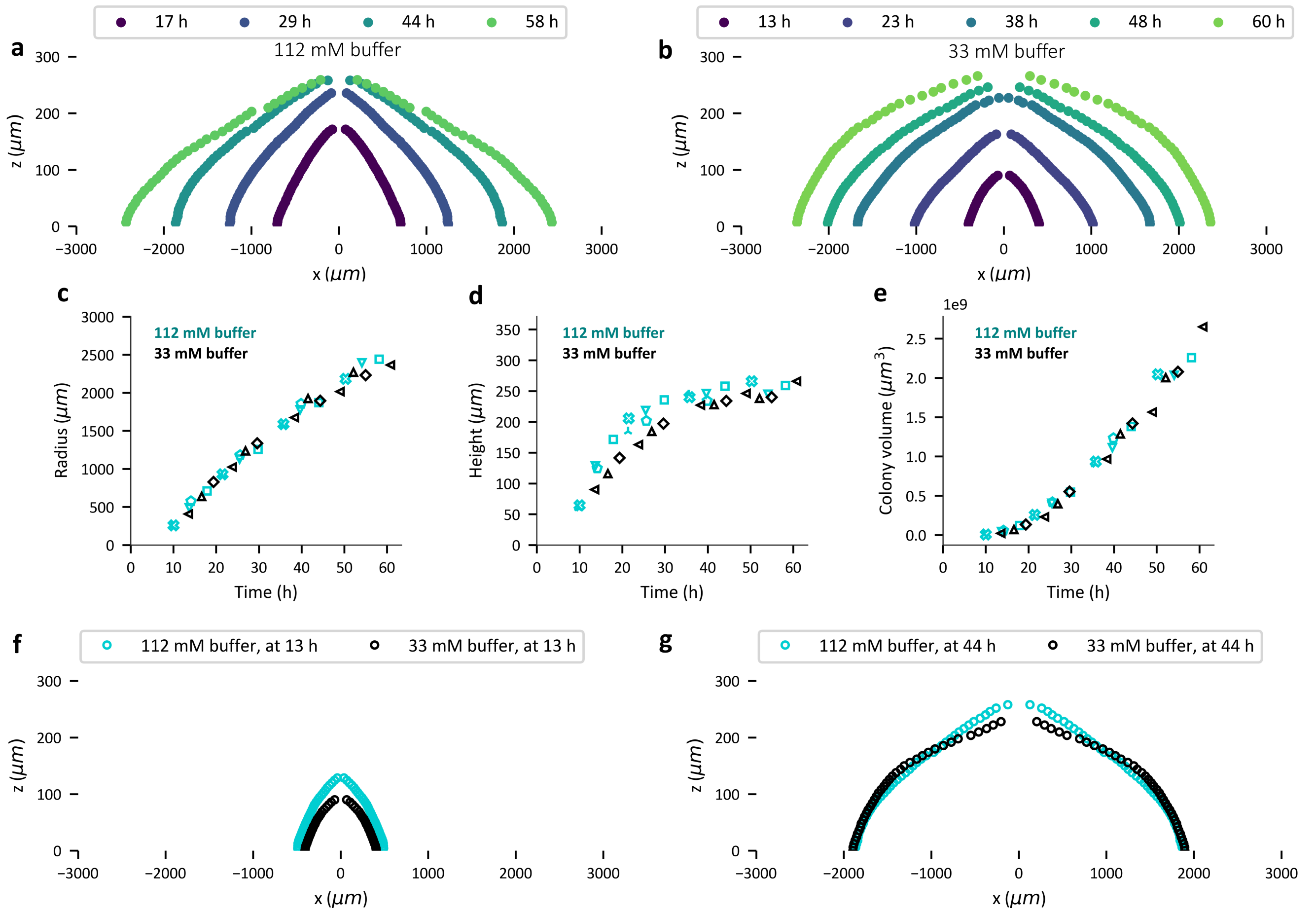

Fig. S9: ***Colony expansion dynamics remains similar when buffer concentration is reduced to 33 mM which indicates lack of acid stress with 112 mM buffer.*** Expansion dynamics of EQ59 *E. coli* colonies on 1.5 % (w/v) agar plates prepared with 20 mM glucose, 10 mM ammonium chloride and a defined concentration (33 mM and 112 mM) of phosphate buffer at various times post-inoculation as a single cell. The cross-sectional profile of a colony grown on a minimal media hard agar plate with 112 mM buffer **(a)**, and 33 mM buffer **(b)** at various times (coded by color) post-inoculation. The radius (μm) **(c)**, height (μm) **(d)**, and volume (μm^3) **(e)** of the colonies plotted against the time (h) post-inoculation. Comparison of cross-section profile of a colony grown with 112 mM buffer and 33 mM buffer at $\sim$13 h **(f)**, and $\sim$44 h **(g)**. Cyan symbols represent colonies grown on minimal media plates with 112 mM buffer while black symbols represent colonies with 33 mM buffer. For (c-e) each type of symbol represents an individual biological replicate. Results shown here for the 112 mM buffer condition correspond to the same data presented for the 20 mM glucose condition in Fig. 1 of main text.

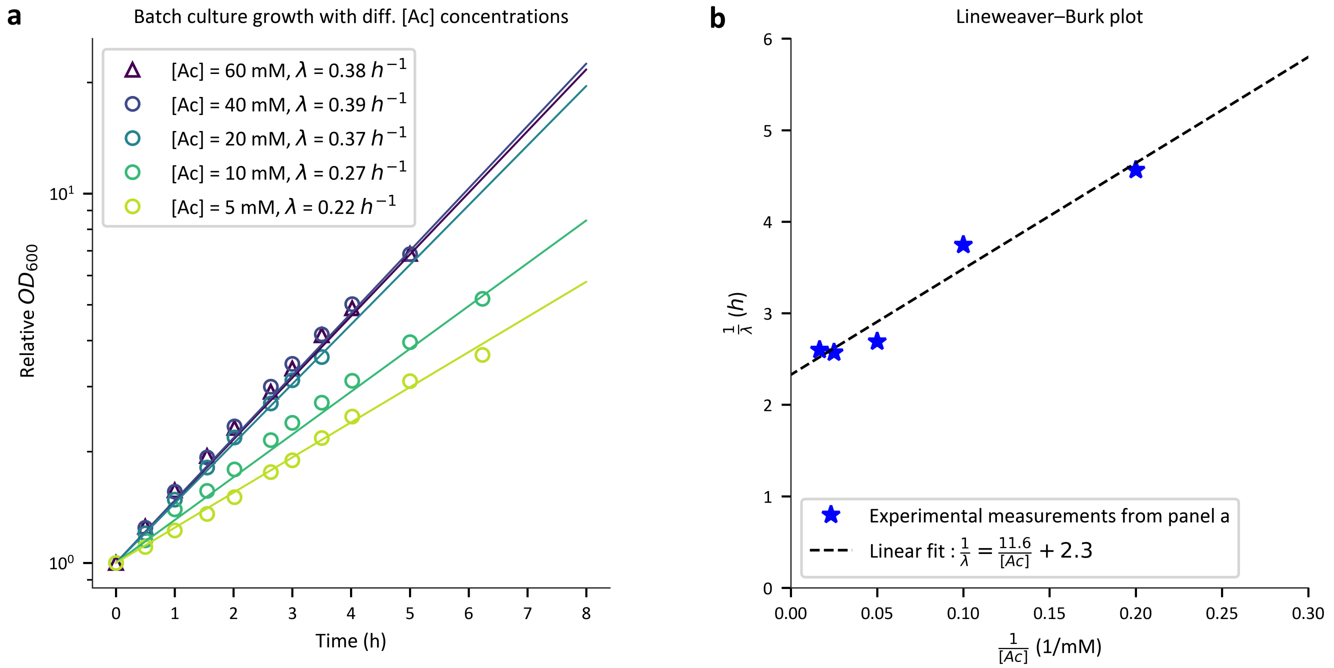

Fig. S10: **(a)** Batch culture growth curves when EQ59 is grown with a defined concentration ranging from 5 mM to 60 mM of sodium acetate (coded by color) as the sole carbon source, 10 mM ammonium chloride and 112 mM phosphate buffered minimal medium. **(b)** Lineweaver-Burk plot of reciprocal of growth rate vs. the reciprocal of acetate concentration (blue stars) where the growth rate for each condition is obtained by fitting data in panel (a) to an exponential function. Black dashed lines represent the best fit of data to a function of the form $\frac{1}{\lambda} = m\frac{1}{[\text{Ac}]} + c$. Assuming a Monod form for the growth rate dependence on acetate concentration i.e., $\lambda([\text{Ac}]) = \frac{\lambda_{max}[\text{Ac}]}{[Ac]+K_a}$, then note that $c = \frac{1}{\lambda_{max}}$ and $K_a = \frac{m}{c}$. Based on the best linear fit (plotted in black dashed lines) $K_a \approx$ 5 mM and $\lambda_{max} \approx 0.4\, h^{-1}$.

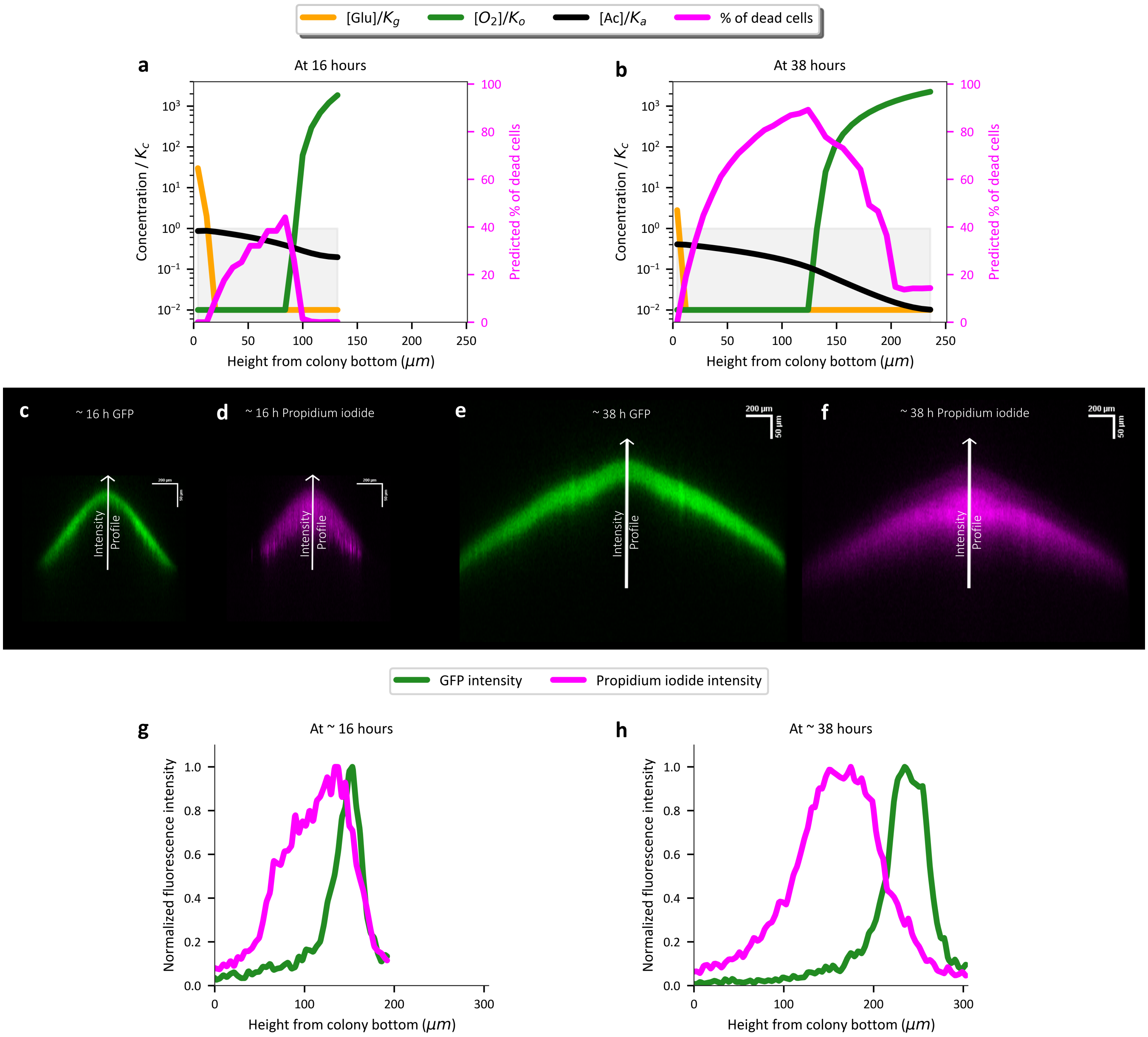

Fig. S11: ***Death zone is localized to the colony interior.*** **(a-b)** Left y-axis : Glucose (yellow), oxygen (green) and acetate (black) concentration normalized by respective Monod constants K_g, K_o, and K_a along the central vertical axis of the simulated colony plotted against the vertical distance from colony bottom (µm) at 16 h stage **(a)**, and 38 h **(b)** of simulated colony development. The height of the simulated colony at 16 h and 38 h was 136 µm and 242 µm respectively. Right y-axis: The predicted % of dead cells (magenta) along the central vertical axis of the simulated colony is plotted against the vertical distance from colony bottom (µm). **c-d** GFP fluorescence (in green) and propidium iodide fluorescence (colored with a magenta LUT) in an optical cross-section at the center of a $\sim$16 h old colony. **e-f** GFP fluorescence (in green) and propidium iodide fluorescence (colored with a magenta LUT) in an optical cross-section at the center of a $\sim$38 h old colony. Images shown in panels c-f represent the same data shown in Fig. 7ij of main text. The vertical line represents the line along which the fluorescence internsity is reported in panels g-h. **(g-h)** GFP intensity (in green) and propidium iodide fluorescence intensity (in magenta) normalized by the respective maximum intensities along the central vertical axis (see panels c-f) of an optical cross-section at the center (i.e., (0,0,z) axis) of a $\sim$16 h old **(g)**, and $\sim$38 h old **(h)** experimental colony obtained using two-photon microscopy. Simulation and experimental results presented here correspond to 20 mM initial glucose concentration. Data shown in panels c-h here are for the same colony represented in Fig. 7g-j of main text.

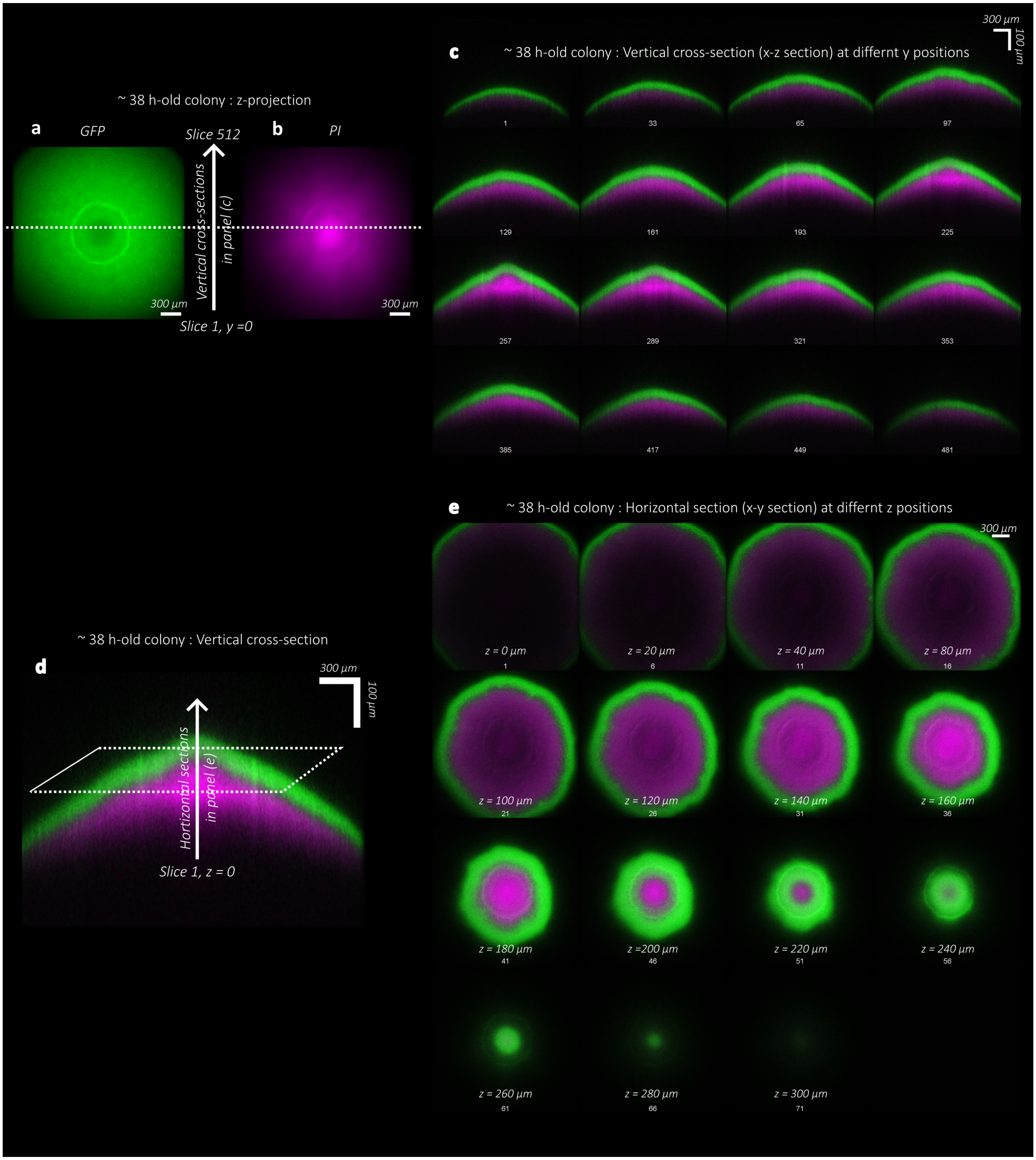

Fig. S12: ***PI fluorescence along cross-sections of a 2 day-old colony.*** Summed intensity z-projection of GFP fluorescence intensity (in green) **(a)**, and Propidium Iodide (PI) fluorescence (in magenta) **(b)** of a ∼38 h old colony obtained using two-photon microscopy (see Methods). **(c)** Merged images of GFP fluorescence (in green) and Propidium iodide fluorescence (in magenta) along the various vertical cross-sections of a ∼38 h-old colony. **(d)** Merged GFP (green) and PI (magenta) fluroescence along the vertical cross-section at the center of a ∼38 h old colony. **(e)** Merged images of GFP fluorescence (in green) and Propidium iodide fluorescence (in magenta) along the various horizontal-sections of a ∼38 h-old colony. The images of the colony in this figure represent the same colony whose data is shown in Fig. 7h,j of main text.

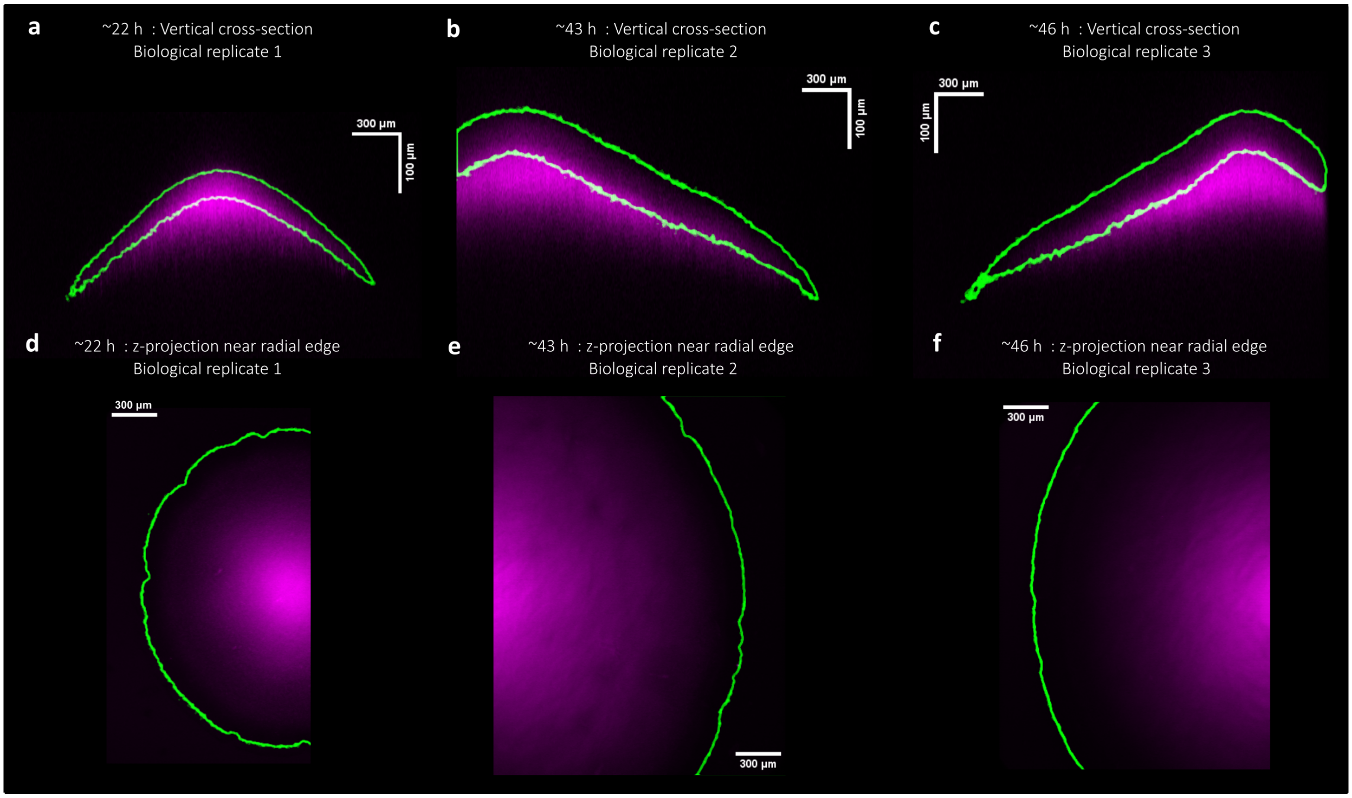

Fig. S13: ***Biological replicates for detecting localization of death zone.*** **(a-c)** Propidium Iodide (PI) fluorescence (in magenta) along the vertical cross-section at the center of a $\sim$22 h old colony **(a)**, a $\sim$43 h old colony **(b)**, and a $\sim$46 h old colony **(c)** obtained using two-photon microscopy. The three colonies represented in panels a,b,c represent three individual biological replicates respectively. **(d-f)** Summed intensity z-projection of Propidium Iodide (PI) fluorescence (in magenta) near the radial edge of the colonies shown in panels a-c, i.e., a $\sim$22 h old colony **(d)**, a $\sim$43 h old colony **(e)**, and a $\sim$46 h old colony **(f)** obtained using two-photon microscopy. Colonies shown in this figure were grown in 20 mM glucose minimal media plates (see Methods). In panels a through f, the outline of the corresponding signal from GFP fluorescence is represented in green.

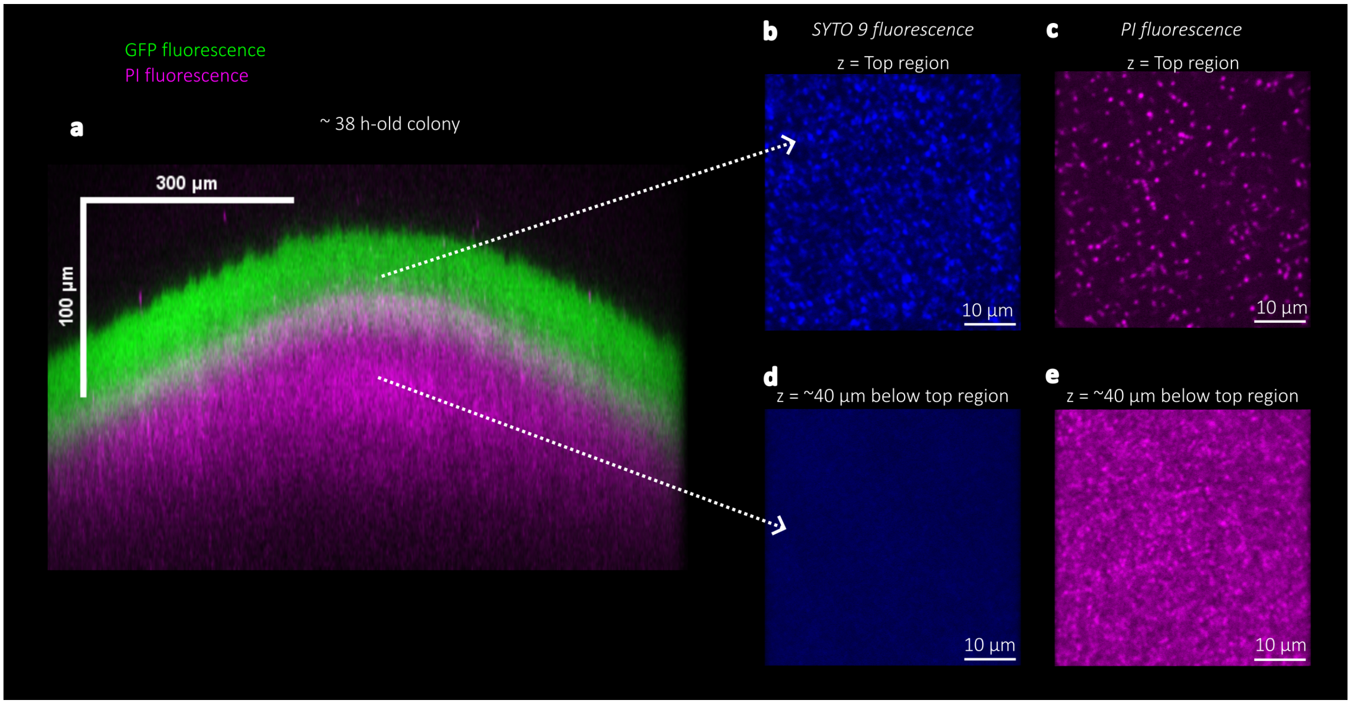

Fig. S14: ***High resolution images of PI fluorescence along cross-sections of a ∼2 day-old colony.*** **(a)** Merged GFP (green) and PI (magenta) fluroescence along the vertical cross-section at the center of a ∼38 h old EQ59 colony obtained with two-photon microscopy using a 25x/1 NA water immersion objective (25x/1 NA; Olympus, XLPLN25XSVMP2). A thin layer of low-melt agarose was poured carefully at ∼37 degree Celcius onto the agar plate with the colony and solidified few minutes before imaging to permit the use of water immersion objective to image the colony from the top in an upright position. **(b-e)** Zoomed-in images (using water immersion objective) of a central cross-section of a ∼38 h-old EQ54 colony displaying SYTO 9 fluorescence (in blue) and propidium iodide fluorescence (in magenta) at two different z-positions. The colony was grown on 20 mM glucose minimal media agar plates with equal concentration (2.5 µM) of STYO 9 and propidium iodide (LIVE/DEAD™ BacLight™ Bacterial Viability Kits, Thermo Fisher Catalog No. L7012). Per manufacturer's documentation, SYTO 9 stain generally labels all cells while propidium iodide labels cells with damaged membranes. When both stains are present simulataneously, the prescence of PI causes a reduction in SYTO 9 stain fluorescence. Thus, cells with blue are indicative of cells with intact membranes (presumed alive) and cells with magenta are indicative of cells with damaged membrane (presumed dead). The colony represented in panels b-e is that of EQ54 strain (the parent strain of EQ59) which does not express GFP. The reason EQ54 was used is due to the overlapping emission spectrum of GFP and SYTO 9 fluorescence (FITC) and thus we required a strain that does not express GFP for this experiment. The EQ59 colony represented in panel a was grown parallely in an identical agar plate (expect without SYTO 9) and was inoculated and imaged together. The white arrows represent the approximate z-regions that panels b,c and d,e represent along the colony's vertical cross-section.

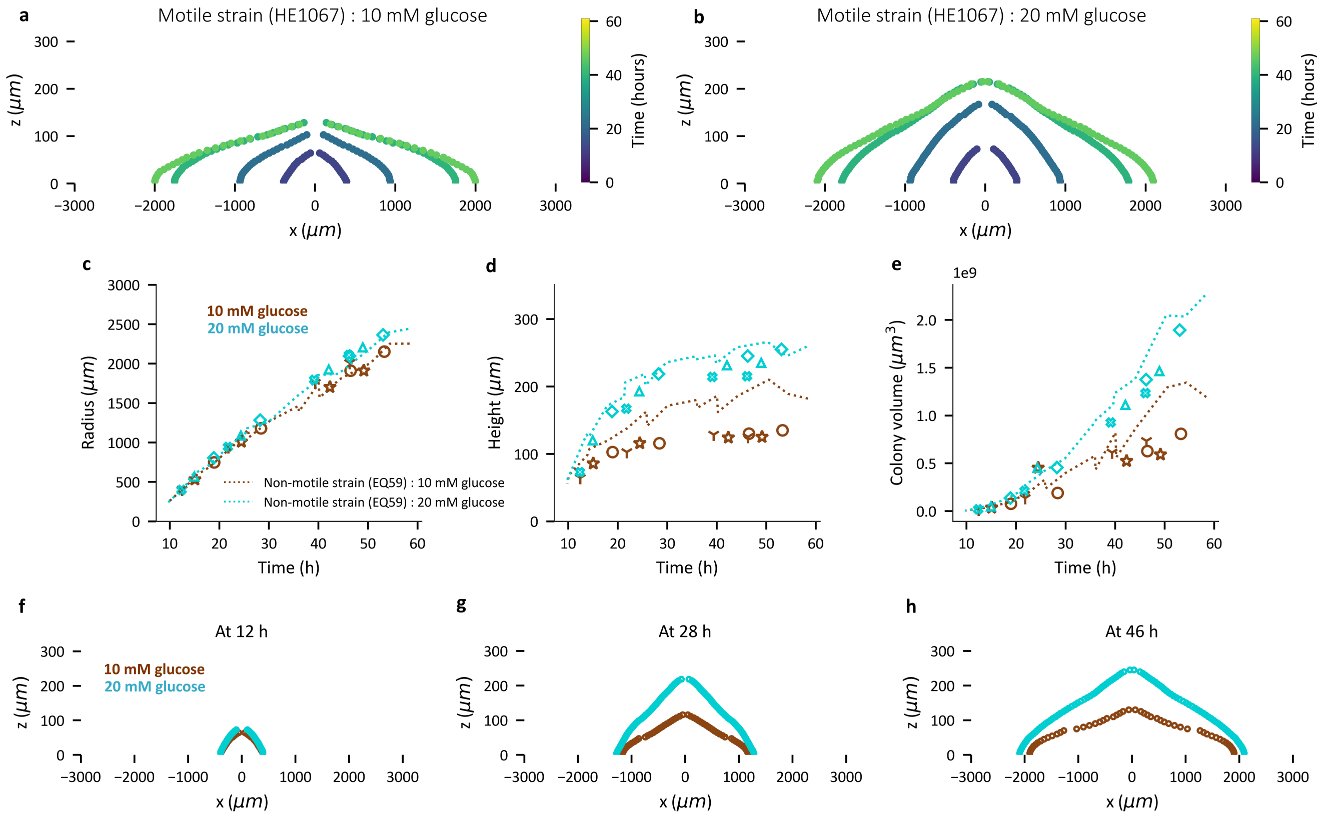

Fig. S15: ***Effect of initial glucose concentration on colony expansion of a motile E. coli strain.*** Expansion dynamics of colonies formed by a motile *E. coli* strain - HE1067 on 1.5 % (w/v) hard agar plates prepared with defined concentration of glucose, 10 mM ammonium chloride concentration, and 112 mM phosphate buffer at various times post-inoculation as a single cell. The cross-sectional profile of a colony grown on a minimal media hard agar plate with 10 mM glucose **(a)** and 20 mM glucose **(b)** shown for various times (coded by color) post-inoculation. The radius (μm) **(c)**, height (μm) **(d)**, and volume (μm^3) **(e)** of the HE1067 colonies plotted against the time (h) post-inoculation. Brown symbols represent colonies grown on minimal media plates with 10 mM glucose while cyan symbols represent colonies with 20 mM glucose. Each shape of symbol represents an individual biological replicate. The dotted lines represent data shown in Fig. 1 for EQ59 (non-motile) colonies grown in 10 mM glucose (brown) and 20 mM glucose (cyan) minimal media agar plates. Comparison of the cross-sectional profiles of HE1067 colonies grown with 10 mM glucose (brown) and 20 mM glucose (cyan) at $\sim$12 h **(f)**, $\sim$28 h **(g)** and $\sim$46 h post-inoculation **(h)**. It is seen that similar to the observations for non-motile EQ59 colonies (Fig. 1 in main text), radial expansion of motile HE1067 colonies remains similar for 10 mM and 20 mM glucose concentration, while vertical expansion increases when the glucose concentration is increased to 20 mM. In general, the height of colonies formed by the motile strain (HE1067) are lower than the colonies formed by the non-motile strain (EQ59) for either glucose concentrations. This could presumably arise due to an elevated energy demand caused by flagella expression/rotation in the motile strain (1) which would result in higher glucose consumption by cells, thereby worsening glucose depletion inside the colony. However, despite quantitative differences in colony height between non-motile and motile strains, the effect of glucose concentration on colony expansion remains similar qualitatively.

Supplementary Notes

1 - Supplementary Note 1: An Agent-Based Model for Cell Activities

The agent-based component of our model to simulate the growth, division, and movement of individual bacterial cells within a colony growing on agar is adapted from Warren et.al. (2). Here, we provide a brief overview of the key model components.

1.1 - Cell growth, division, and movement

We model an *E. coli* cell as a sphero-cylindrical agent, i.e., a cylinder with hemispherical caps on both ends. The length of the cylinder, $l,$ elongates during cell growth, while the diameter, $w_0,$ of the hemispherical caps (also referred to as the cell width) remains constant during the cell growth. We denote by $\vec{p}$ and $\vec{q}$ the position vectors of the centers of the two hemispheres, by $\ell = \|\vec{p} - \vec{q}\|$ the cell cylindrical length, and by $\vec{n} = (\vec{q} - \vec{p})/\ell$ the unit vector pointing from one center of hemisphere $\vec{p}$ to the other $\vec{q}$ and refer to it as the *director* of the cell. The center of mass for the cell is taken to be $\vec{r}_{\text{c}} = (\vec{p} + \vec{q})/2.$

The length $\ell = \ell(t)$ of a cell increases at the cell elongation rate $\tilde{\lambda}(t)$ with time t. The elongation rate is proportional to the mass growth rate $\lambda(t)$ of the cell, i.e., $\tilde{\lambda}(t) = \sigma\lambda(t)$, where the proportionality factor is $\sigma = \ln 3/\ln 2$ (2). The mass growth rate of a cell at a particular time t is calculated as $\lambda(t) = \lambda(\vec{r}_{\text{c}}(t), t)$, where $\vec{r}_{\text{c}}(t)$ is the center of the cell at time t and $\lambda(\vec{r}, t)$ is the local growth rate at spatial point $\vec{r}$ and time t. The local growth rate is determined by the local concentrations of glucose, oxygen, and acetate (see Section 2.1 for details).

Each cell starts off with the same cylindrical length ℓ_0 and grows with the elongation of its cylindrical length $\ell(t)$ governed by the growth equation,

$$\dot{\ell}(t) = \sigma\lambda(\vec{r}_{\text{c}}(t), t)\ell(t),$$

where $\dot{\ell}(t)$ denotes the time derivative of $\ell(t)$. Numerically, we update the cell length from time t to $t + \Delta t$ by

$$\ell(t + \Delta t) = \ell(t) + \sigma\lambda(\vec{r}_{\text{c}}(t), t)\ell(t)\Delta t.$$

Once the cylindrical length $\ell(t)$ increases by a ΔL amount, by the adder principle (3), the cell divides into two daughter cells. Upon division, the two daughter cells inherit the velocity and angular velocity from their mother cell with some fluctuations in their angular velocities.

The position and orientation of a cell changes according to its velocity $\vec{v}$ and angular velocity $\vec{\omega}$, which follow Newton's second law

$$M\frac{\partial \vec{v}}{\partial t} = \vec{F}^{\text{net}} \qquad \text{and} \qquad I\frac{\partial \vec{\omega}}{\partial t} = \vec{T}^{\text{net}}, \tag{1}$$

where M and I are the mass and moment of inertia of the cell, and $\vec{F}^{\text{net}}$ and $\vec{T}^{\text{net}}$ are the net force and net torque, respectively, exerted on that cell. A brief description of the forces exerted on a cell is provided in the next subsection. Once the net force and torque are calculated, following the numerical scheme used in (2), we use the velocity-Verlet algorithm (4) to update individual cell positions, orientations, velocities, and angular velocities based on Newton's law (Eq. [1]).

1.2 - Interaction forces

The forces and torques exerted on a cell within the simulated colony arise from the following four factors:

(1) **Cell-cell mechanical interaction:**
Following the implementation of Warren et.al. (2019) (2), two cells in contact with each other generate a contact force that is described by its components along the normal and tangential direction. The normal component of this force is the sum of the Hertz contact force component $\propto \sqrt{w_0}\delta_{\text{cc}}^{3/2}$ (where w_0 is the cell-width, δ_{cc} is the amount of overlap of the two cells), and the normal component of a dissipation force with magnitude $\propto \delta_{\text{cc}}^{1/2} v_{\text{cc},n}$ (where $v_{\text{cc},n}$ is the normal component of the cell-cell relative velocity of the two cells). The tangential force component is taken to be the minimum of the tangential dissipation force component

$\propto \delta_{cc} v_{cc,t}$ (where $v_{cc,t}$ is the tangential component of the cell-cell relative velocity of the two cells) and the Hertz contact force multiplied by the *dynamic* friction coefficient (2). It should be noted that in high-density colonies such as those studied here, dissipation due to viscous drag is significantly less than the cellular friction force.

(2) **Cell-agar interaction** (if the cell is in contact with the agar surface):
The cell-agar interaction force is modeled as a contact force with elastic and frictional components similar to the cell-cell interaction force described above.

(3) **Cell-fluid interaction:**
The cell-fluid interaction force is modeled as a Stokes drag force which is proportional to the cell velocity.

(4) **Surface tension** (if the cell is at the colony-air boundary):
Surface tension between the air and liquid molecules coating a bacterial cell arises when the cell protrudes out on the top of bacterial colony, increasing the air-liquid interfacial area, thereby resulting in a restoring force exerted on the cell. The surface tension is implemented as a boundary force, i.e., as a force experienced by discrete cells at the colony boundary due to increased surface tension of the continuum liquid these cells are immersed in. As studied in Warren et al. (2019) (2), this individual cell-level implementation of surface tension can hold a large group of cells as a monolayer above the agar surface, until the pressure inside the expanding monolayer (due to friction against motion on agar surface) exceeds a critical level. At this critical level the pressure build up overcomes the surface tension resisting vertical protrusion of cells, thereby resulting in the 'buckling' of the monolayer of cells into multiple layers. This buckling of cells marks the transition from a 2D monolayer colony into a 3D colony.

2 - Supplementary Note 2: A Continuum Model for Metabolism

2.1 - Metabolic model to determine cell growth rate

The following three metabolic processes contribute to cell growth in our model:

(a) Aerobic growth on glucose: Cells consume glucose and oxygen, and produce biomass and excrete acetate.

$$\text{Glucose} + \text{Oxygen} \rightarrow \text{Biomass} + \text{Acetate};$$

(b) Anaerobic growth on glucose: Cells consume glucose in the absence of oxygen, and produce biomass and excrete acetate.

$$\text{Glucose} \rightarrow \text{Biomass} + \text{Acetate};$$

(c) Aerobic growth on acetate: Cells consume acetate and oxygen, and produce biomass.

$$\text{Acetate} + \text{Oxygen} \rightarrow \text{Biomass}.$$

We use appropriately modified Monod kinetic forms to determine the local cell growth rate as a function of the local concentrations of glucose $C_g = C_g(\vec{r}, t)$, acetate $C_a = C_a(\vec{r}, t)$, and oxygen $C_o = C_o(\vec{r}, t)$. First, we define for convenience,

$$\theta_g = \frac{C_g}{C_g + K_g}, \qquad \theta_a = \frac{C_a}{C_a + K_a}, \qquad \theta_o = \frac{C_o}{C_o + K_o}, \qquad [2]$$

where K_g, K_a, and K_o are the Monod constants corresponding to glucose, acetate and oxygen uptake by cells, respectively. We then determine the local cell growth rate $\lambda = \lambda(\vec{r}, t)$ at spatial point $\vec{r}$ in the colony and time t by,

$$\lambda = \underbrace{\lambda_1(\lambda_{g,aer}, C_g)\theta_o}_{\text{Aerobic growth on glucose}} + \underbrace{\lambda_2(\lambda_{g,ana}, C_g)(1-\theta_o)}_{\text{Anaerobic growth on glucose}} + \underbrace{\lambda_3(\lambda_{a,aer}, C_a)(1-\theta_g)\theta_o}_{\text{Aerobic growth on acetate}}. \qquad [3]$$

Here, the factors θ_o and $(1-\theta_o)$ serve as weights to decide whether cells are under aerobic conditions (i.e., $\theta_o = 1$) or anaerobic conditions (i.e., $\theta_o = 0$ which implies $1-\theta_o = 1$). Further, under aerobic conditions, the factor $(1-\theta_g)$ is used to determine whether cells grow on glucose or acetate, i.e., to model the hierarchical preference of glucose by *E. coli* for growth. In other words, if glucose concentration is saturating, then $\theta_g = 1$ and $1 - \theta_g = 0$. Hence, the component of growth rate arising from acetate is 0. In a model which does not account for cell maintenance, the subscripted λ-terms would traditionally be defined using Monod kinetic forms, i.e.,

$$\lambda_1(\lambda_{g,aer}, C_g) = \lambda_{g,aer}\theta_g, \quad \lambda_2(\lambda_{g,ana}, C_g) = \lambda_{g,ana}\theta_g, \quad \lambda_3(\lambda_{a,aer}, C_a) = \lambda_{a,aer}\theta_a, \qquad [4]$$

where, $\lambda_{g,aer}$, $\lambda_{g,ana}$, and $\lambda_{a,aer}$ are the maximum growth rates possible under aerobic growth on glucose, anaerobic growth on glucose, and aerobic growth on acetate, respectively. However, to incorporate the effects of cell maintenance we appropriately modify the local growth rates (see Section 2.3). Note that this growth model described by Eq. [3] serves as a tri-state switch, i.e., when both glucose and oxygen are abundant, aerobic growth on glucose will turn on; when oxygen is lacking, anaerobic growth on glucose will turn on; and when glucose is lacking, aerobic growth on acetate will turn on.

2.2 - Reaction-diffusion model for spatiotemporal dynamics of nutrients in (1+1)-dimensions

The geometry of our model for a bacterial colony growing on hard agar is illustrated in Fig. S16. The computational domain which is (1+1)-dimensional, $\Omega = (-L, L) \times (-a, b)$, includes the air region Ω_0 (colored blue), the colony region Ω_1 (colored salmon), and the agar region Ω_2 (colored yellow). We denote by Γ_{01} the interface separating the air region Ω_0 and the colony region Ω_1, by Γ_{02} the interface separating the air region Ω_0 and the agar region Ω_2, and by Γ_{12} the interface that separates the agar region Ω_1 and colony region Ω_2. The lateral and bottom faces of the agar region Ω_2 are marked by Γ_s and Γ_b, respectively. Since the bacterial colony grows with time t, all the air region Ω_0, the colony region Ω_1, the colony-air interface Γ_{01}, and the colony-agar interface Γ_{02} depend on time

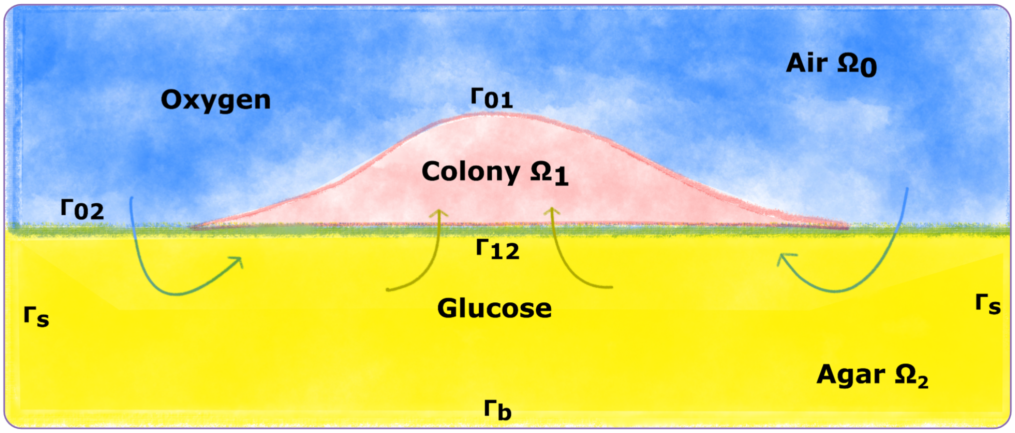

Fig. S16: An illustration of the (1+1)-dimensional computational domain and the different sub-regions involved in the simulations of colony growth. The overall region is $\Omega = (-L, L) \times (-a, b)$, where all L, a, and b are positive numbers in the units of length. It is divided into the air region Ω_0, colony region Ω_1, and agar region $\Omega_2 = (-L, L) \times (-a, 0)$, respectively. The colony surface or colony-air interface Γ_{01} separates the colony from air. The plane $z = 0$ in the system region is divided into two parts. One is the interface that separates the colony from agar, and is denoted by Γ_{12}. The other part, denoted Γ_{02}, separates the air from agar. Note that, since the bacterial colony grows with time t, the air region Ω_0, the colony region Ω_1, the colony-air interface Γ_{01}, and the colony-agar interface Γ_{02}, all depend on time t.

t. Note that oxygen in the air region Ω_0 diffuses into the bacterial colony and that glucose in agar diffuses into the colony and taken up by bacterial cells.

A system of reaction-diffusion partial differential equations (PDEs) for metabolite concentrations is used to model the spatiotemporal dynamics of metabolism within the colony. The concentrations of glucose $C_g = C_g(\vec{r}, t)$, acetate $C_a = C_a(\vec{r}, t)$, and oxygen $C_o = C_o(\vec{r}, t)$ are all defined spatially in both the colony region Ω_1 which expands with time and the agar region Ω_2 that is fixed throughout the simulation. For convenience, we shall also denote the colony and agar regions by Ω_+ (same as Ω_1) and Ω_- (same as Ω_2), respectively.

Glucose. The reaction-diffusion equations for the glucose concentration are given by

$$\frac{\partial C_g}{\partial \mathrm{t}} = D_{g,-} \Delta C_g \qquad \text{in agar region } \Omega_-, \qquad [5]$$

$$\frac{\partial C_g}{\partial \mathrm{t}} = D_{g,+} \Delta C_g - \rho Q_g \qquad \text{in colony region } \Omega_+. \qquad [6]$$

Here, $D_{g,-}$ and $D_{g,+}$ are the diffusion coefficients for glucose in agar and colony, respectively, ρ is the local cell density within the colony, and Q_g is the glucose consumption rate. We model such a rate with Monod kinetics:

$$Q_g = \underbrace{q_{g,aer} \lambda_1(\lambda_{g,aer}, C_g) \theta_o}_{\text{Aerobic glucose consumption}} + \underbrace{q_{g,ana} \lambda_2(\lambda_{g,ana}, C_g)(1 - \theta_o)}_{\text{Anaerobic glucose consumption}}, \qquad [7]$$

where $q_{g,aer}$ and $q_{g,ana}$ denote the specific glucose uptake fluxes under aerobic and anaerobic growth conditions, respectively, and the λ_1-term and λ_2-term are the local growth rates for aerobic growth on glucose and anaerobic growth on glucose, respectively.

Equations (5) and (6) are supplemented with the following interface conditions on the colony-agar interface Γ_{12} that couple the glucose concentration $C_{g,-}$ from the agar region and $C_{g,+}$ from the agar region:

$$C_{g,-} = C_{g,+} \quad \text{and} \quad D_{g,+} \frac{\partial C_{g,+}}{\partial z} = D_{g,-} \frac{\partial C_{g,-}}{\partial z} \quad \text{on colony-agar interface } \Gamma_{12}.$$

The boundary conditions for the glucose concentration are

$$\frac{\partial C_g}{\partial n} = 0 \qquad \text{on } \Gamma_s \cup \Gamma_b \cup \Gamma_{02} \cup \Gamma_{01},$$

where $\partial/\partial n$ denotes the normal derivative.

Given an initial value of the glucose concentration, the system of these reaction-diffusion equations, together with the interface conditions and boundary conditions, determine uniquely the spatiotemporal dynamics of the glucose. In our simulations, we set the initial value of glucose to be a constant, $C_{g,0}$, in the agar region Ω_- but 0 in the colony region Ω_+.

Oxygen. The reaction-diffusion equations for the oxygen concentration are given by

$$\frac{\partial C_o}{\partial \mathrm{t}} = D_{o,-}\Delta C_o \qquad \text{in agar region } \Omega_-, \qquad [8]$$

$$\frac{\partial C_o}{\partial \mathrm{t}} = D_{o,+}\Delta C_o - \rho Q_o \qquad \text{in colony region } \Omega_+, \qquad [9]$$

where $D_{o,-}$ and $D_{o,+}$ are the diffusion coefficients for oxygen in agar and colony, respectively, and Q_o is the oxygen consumption rate. We take the following form of this rate:

$$Q_o = \underbrace{q_{o,g}\lambda_1(\lambda_{g,aer}, C_g)\theta_o}_{\text{Oxygen uptake during growth on glucose}} + \underbrace{q_{o,a}\lambda_3(\lambda_{a,aer}, C_a)(1-\theta_g)\theta_o}_{\text{Oxygen uptake during growth on acetate}}, \qquad [10]$$

where $q_{o,g}$ and $q_{o,a}$ are the specific uptake fluxes of oxygen during the aerobic growth on glucose and that on acetate, respectively, and $\lambda_3(\lambda_{a,aer}, C_a)$ is the growth rate for aerobic growth on acetate, given in Eq. (4).

The interface conditions for the oxygen concentration on the colony-agar interface that couple the oxygen concentration $C_{o,-}$ in the agar and $C_{o,+}$ in the colony are given by

$$C_{o,-} = C_{o,+} \quad \text{and} \quad D_{o,+}\frac{\partial C_{o,+}}{\partial z} = D_{o,-}\frac{\partial C_{o,-}}{\partial z} \quad \text{on colony-agar interface } \Gamma_{12}.$$

The boundary conditions for the oxygen concentrations are,

$$C_o = C_{o,0} \qquad \text{on } \Gamma_{01} \cup \Gamma_{02},$$

$$\frac{\partial C_o}{\partial n} = 0 \qquad \text{on } \Gamma_s \cup \Gamma_b,$$

where $C_{o,0}$ is the boundary value of oxygen concentration which is taken to be a constant of 0.26 mM, approximately the concentration of oxygen in air. We set the initial oxygen concentration to be 0.26 mM in the agar region. Note that our choice for the boundary value and initial value of oxygen concentration assumes equilibration between air and agar.

Acetate. The reaction-diffusion equations for the acetate concentration are given by

$$\frac{\partial C_a}{\partial \mathrm{t}} = D_{a,-}\Delta C_a \qquad \text{in agar region } \Omega_-, \qquad [11]$$

$$\frac{\partial C_a}{\partial \mathrm{t}} = D_{a,+}\Delta C_a + \rho P_a - \rho Q_a \qquad \text{in colony region } \Omega_+, \qquad [12]$$

where $D_{a,-}$ and $D_{a,+}$ are the diffusion coefficients for acetate in agar and colony, respectively, P_a is the acetate excretion rate during cell growth on glucose, and Q_a is the acetate consumption rate during the cell growth on acetate. We model these rates using appropriate Monod kinetic forms as follows:

$$P_a = \underbrace{p_{a,aer}\lambda_1(\lambda_{g,aer}, C_g)\theta_o}_{\text{Aceate production during aerobic growth}} + \underbrace{p_{a,ana}\lambda_2(\lambda_{g,ana}, C_g)(1-\theta_o)}_{\text{Acetate production during anaerobic growth}}; \qquad [13]$$

$$Q_a = \underbrace{q_{a,aer}\lambda_3(\lambda_{a,aer}, C_a)(1-\theta_g)\theta_o}_{\text{Acetate consumption}}. \qquad [14]$$

Here, $p_{a,aer}$ and $p_{a,ana}$ denote the specific excretion flux of acetate in the aerobic and anaerobic cell growth on glucose, respectively, $q_{a,aer}$ is the specific uptake flux of acetate during the cell growth on acetate, and all the subscripted λ-terms are growth rates for the three growth modes and are defined in Eq. (4).

The interface conditions for the acetate concentration on the colony-agar interface that couple the acetate concentration $C_{a,-}$ in the agar and $C_{a,+}$ in the colony are given by

$$C_{a,-} = C_{a,+} \quad \text{and} \quad D_{a,+}\frac{\partial C_{a,+}}{\partial z} = D_{a,-}\frac{\partial C_{a,-}}{\partial z} \quad \text{on colony-agar interface } \Gamma_{12}.$$

The boundary conditions for the acetate concentration are (Fig. S16)

$$\frac{\partial C_a}{\partial n} = 0 \qquad \text{on } \Gamma_{01} \cup \Gamma_{02} \cup \Gamma_s \cup \Gamma_b.$$

We set the initial acetate concentration to be 0 in both the agar and colony regions.

2.3 - Model for cell maintenance

Even in the absence of growth, bacterial cells still need energy to perform maintenance activities. Such maintenance requires uptake of a carbon source. Three modes of cell maintenance, namely, maintenance on glucose under aerobic and anaerobic conditions and maintenance on acetate under aerobic conditions are included in our model. Further, with the inclusion of cell maintenance in our model, cells grow only when the nutrient concentration is larger than a threshold value C^* (illustration in Fig. S17).

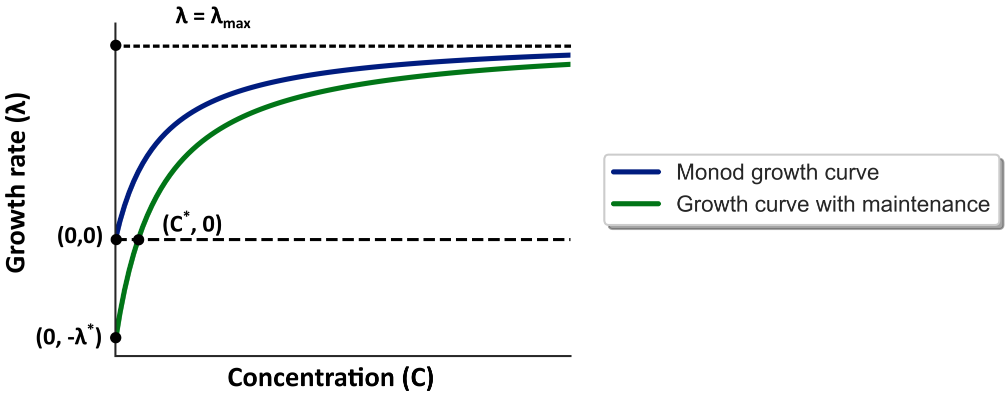

Fig. S17: Dependence of growth rate on nutrient concentration is modified to account for cell maintenance: cells grow only when the nutrient concentration is larger than a threshold value C^*.

Let $q^0_{g,aer}$, $q^0_{g,ana}$, and $q^0_{a,aer}$ denote the maintenance rates for the three modes of maintenance, respectively. Further let,

$$\lambda^*_{g,aer} = \frac{q^0_{g,aer}}{q_{g,aer}}, \qquad \lambda^*_{g,ana} = \frac{q^0_{g,ana}}{q_{g,ana}}, \qquad \lambda^*_{a,aer} = \frac{q^0_{a,aer}}{q_{a,aer}}, \tag{15}$$

where $q_{g,aer}$, $q_{g,ana}$, and $q_{a,aer}$ are the specific uptake and excretion fluxes under various growth conditions which were introduced above in Eqns. (13) and (14). The total growth rate combined across the different modes has the same form as defined in Eq. (3). However, to additionally include the effect of maintenance, we modify the rates λ_1, λ_2, and λ_3 defined in Eq. (4) to be

$$\lambda_1(\lambda_{g,aer}, C_g) = \text{max}\left\{(\lambda_{g,aer} + \lambda^*_{g,aer})\theta_g - \lambda^*_{g,aer},\ 0\right\}, \tag{16}$$
$$\lambda_2(\lambda_{g,ana}, C_g) = \text{max}\left\{(\lambda_{g,ana} + \lambda^*_{g,ana})\theta_g - \lambda^*_{g,ana},\ 0\right\}, \tag{17}$$
$$\lambda_3(\lambda_{a,aer}, C_a) = \text{max}\left\{(\lambda_{a,aer} + \lambda^*_{a,aer})\theta_a - \lambda^*_{a,aer},\ 0\right\}. \tag{18}$$

The threshold nutrient concentration (see Fig. S17) for the three growth modes, namely, $C^*_{g,aer}$, $C^*_{g,ana}$, and $C^*_{a,aer}$ are defined by

$$\lambda_1(\lambda_{g,aer}, C^*_{g,aer}) = 0, \qquad \lambda_2(\lambda_{g,ana}, C^*_{g,ana}) = 0, \qquad \lambda_3(\lambda_{a,aer}, C^*_{a,aer}) = 0.$$

This threshold concentration represents the local nutrient (glucose or acetate) concentration at which the maintenance rate corresponding to the particular mode can be sustained. Beyond these threshold concentrations, the cell exhibits growth under the corresponding growth mode. Solving for $C^*_{g,aer}$, $C^*_{g,ana}$, and $C^*_{a,aer}$, we obtain

$$C^*_{g,aer} = K_g \frac{\lambda^*_{g,aer}}{\lambda_{g,aer}}, \qquad C^*_{g,ana} = K_g \frac{\lambda^*_{g,ana}}{\lambda_{g,ana}}, \qquad C^*_{a,aer} = K_a \frac{\lambda^*_{a,aer}}{\lambda_{a,aer}}.$$

Consequently, we can rewrite [16]–[18] as

$$\lambda_1(\lambda_{g,aer}, C_g) = \begin{cases} (\lambda_{g,aer} + \lambda^*_{g,aer})\theta_g - \lambda^*_{g,aer}, & \text{if } C_g > C^*_{g,aer} \\ 0 & \text{if } C_g \leq C^*_{g,aer} \end{cases},$$

$$\lambda_2(\lambda_{g,ana}, C_g) = \begin{cases} (\lambda_{g,ana} + \lambda^*_{g,ana})\theta_g - \lambda^*_{g,ana}, & \text{if } C_g > C^*_{g,ana} \\ 0 & \text{if } C_g \leq C^*_{g,ana} \end{cases},$$

$$\lambda_3(\lambda_{a,aer}, C_a) = \begin{cases} (\lambda_{a,aer} + \lambda^*_{a,aer})\theta_a - \lambda^*_{a,aer}, & \text{if } C_a > C^*_{a,aer} \\ 0 & \text{if } C_a \leq C^*_{a,aer} \end{cases}.$$

The form of the overall local growth rate at a given spatial location is still the same as given in Eq. (3), i.e. ,

$$\lambda = \lambda_1(\lambda_{g,aer}, C_g)\theta_o + \lambda_2(\lambda_{g,ana}, C_g)(1-\theta_o) + \lambda_3(\lambda_{g,aer}, C_a)(1-\theta_g)\theta_o. \qquad [19]$$

Further, to account for the uptake of nutrients due to cell maintenance activities, we modify the uptake and excretion rates Q_g, Q_o, P_a, and Q_a, defined in Eqns. (7), (10), (13), and (14) to be

$$Q^M_g = Q_g + q^0_{g,aer}\tau(C_g, C^*_{g,aer})\,\theta_o + q^0_{g,ana}\tau(C_g, C^*_{g,ana})\,(1-\theta_o), \qquad [20]$$

$$Q^M_o = Q_o + q^0_{o,g}\tau(C_g, C^*_{g,aer})\,\theta_o + q^0_{o,a}\tau(C_a, C^*_{a,aer})\,(1-\theta_g)\theta_o, \qquad [21]$$

$$P^M_a = P_a + p^0_{a,ana}\tau(C_g, C^*_{g,ana})\,(1-\theta_o), \qquad [22]$$

$$Q^M_a = Q_a + q^0_{a,aer}\tau(C_a, C^*_{a,aer})\,(1-\theta_g)\theta_o, \qquad [23]$$

where, $q^0_{o,g}$ and $q^0_{o,a}$ denote the uptake rate of oxygen during the maintenance on glucose and acetate respectively, and

$$\tau(x,a) = \begin{cases} x/a & \text{if } x \leq a, \\ 1 & \text{if } x > a. \end{cases}$$

The above M-superscripted rates replace Q_g, Q_o, P_a, and Q_a in Eqns. (6), (9), and (12) to incorporate metabolite uptake and acetate excretion during cell-maintenance.

2.4 - Model for tracking nutrient starvation and predicting cell death within colony

The instantaneous uptake flux by a cell for maintenance relative to the maintenance rate corresponding to each of the three maintenance modes, $M^{cell}_{g,aer}(t)$, $M^{cell}_{g,ana}(t)$ and $M^{cell}_{a,aer}(t)$, are defined as

$$M^{cell}_{g,aer}(t) = \tau(C_g, C^*_{g,aer})\theta_o, \qquad [24]$$

$$M^{cell}_{g,ana}(t) = \tau(C_g, C^*_{g,ana})(1-\theta_o), \qquad [25]$$

$$M^{cell}_{a,aer}(t) = \tau(C_a, C^*_{a,aer})(1-\theta_g)\theta_o. \qquad [26]$$

Note that $q^0_{g,aer}\tau(C_g, C^*_{g,aer})\theta_o$, $q^0_{g,ana}\tau(C_g, C^*_{g,ana})(1-\theta_o)$ and $q^0_{a,aer}\tau(C_a, C^*_{a,aer})(1-\theta_g)\theta_o$ are the absolute maintenance uptake rates corresponding to the respective mode of maintenance (as appearing in [20] and [23]). A relative maintenance uptake rate value being x (where $0 \leq x \leq 1$) for a particular maintenance mode indicates

that the nutrient uptake rate of the cell meets $100\ x$% of the corresponding maintenance rate.

We introduce a quantity termed the *deficit* of a cell, $D^{cell}(t)$, to denote the magnitude of a cell's inability to meet the carbon maintenance rate, which is defined by

$$D^{cell}(t) = D^{cell}(t) = \text{max}\left(1 - \left(M^{cell}_{g,aer}(t) + M^{cell}_{g,ana}(t) + M^{cell}_{a,aer}(t)\right)\ ,\ 0\right), \qquad [27]$$

where $M^{cell}_{g,aer}(t)+M^{cell}_{g,ana}(t)+M^{cell}_{a,aer}(t)$ represents the relative carbon uptake flux towards maintenance summed across all three maintenance modes. Thus $D^{cell}(t)$ describes the instantaneous starvation state of a cell where a value of 1 represents complete starvation whereas a value of 0 represents no starvation, and a value between 0 and 1 represents that the maintenance flux is only met partially. Then, the carbon starvation duration, denoted by $\gamma^{cell}(T)$, for a particular cell at time T of colony development is defined by

$$\gamma^{cell}(T) = \int_{\text{Birth time}}^{T} D^{cell}(t)\ dt. \qquad [28]$$

To predict the probability of cell death based on starvation duration it is necessary to differentiate between aerobic starvation duration and anaerobic starvation duration. This is due to our experimental measurements (Fig. 7b of main text) which show that death rate under anaerobic carbon starvation $(\sim 2\ d^{-1})$ is roughly 10-fold higher than the death rate under aerobic carbon starvation $(\sim 0.2\ d^{-1})$. Thus, the instantaneous aerobic deficit $D^{cell}_{aer}(t)$ and anaerobic deficit $D^{cell}_{ana}(t)$ at time t for a particular cell are defined as

$$D^{cell}_{aer}(t) = \begin{cases} \text{max}\left(1 - \left(M^{cell}_{g,aer}(t) + M^{cell}_{a,aer}(t)\right)\ ,\ 0\right) & \text{if } \theta_o > \epsilon \text{ (i.e., in aerobic condition)}, \\ 0 & \text{else}, \end{cases} \qquad [29]$$

$$D^{cell}_{ana}(t) = \begin{cases} \text{max}\left(1 - M^{cell}_{g,ana}(t),\ 0\right) & \text{if } \theta_o \leq \epsilon \text{ (i.e., in anaerobic condition)}, \\ 0 & \text{else}. \end{cases} \qquad [30]$$

Here, ϵ is the threshold to determine whether the cell is under aerobic or anaerobic starvation. In our simulations ϵ is chosen to be 0.01.

Given the instantaneous aerobic and anaerobic deficit of a cell, the aerobic carbon starvation duration $\gamma^{cell}_{aer}(T)$ and anaerobic carbon starvation duration $\gamma^{cell}_{ana}(T)$ at time T of colony development are defined by

$$\gamma^{cell}_{aer}(T) = \int_{\text{Birth time}}^{T} D^{cell}_{aer}(t)\ dt, \qquad [31]$$

$$\gamma^{cell}_{ana}(T) = \int_{\text{Birth time}}^{T} D^{cell}_{ana}(t)\ dt. \qquad [32]$$

Once the aerobic carbon duration $\gamma^{cell}_{aer}(T)$ and anaerobic carbon starvation $\gamma^{cell}_{ana}(T)$ of a particular cell at time T is known, the probability of the cell being dead at time T of colony development is determined by the following equation:

$$\text{Death probability of cell at time } T = \text{max}\left(1 - e^{-\delta_{aer}\gamma^{cell}_{aer}(T)},\ 1 - e^{-\delta_{ana}\gamma^{cell}_{ana}(T)}\right) \qquad [33]$$

where $\delta_{aer} \sim 0.2\ d^{-1}$ is the death rate under aerobic carbon starvation and $\delta_{ana} \sim 2\ d^{-1}$ is the death rate under anaerobic carbon starvation as motivated by our batch culture glucose starvation experiments (Fig. 7b of main text).

3 - Supplementary Note 3: Numerical Methods and Computer Implementation

3.1 - A (1+1)-dimensional approximation

In order to computationally simulate colony expansion till two days (by then the radial dimension is in the order of millimeters and the vertical dimension is hundreds of micrometers), we approximate our underlying colony system with a (1+1)-dimensional geometry. The cells in the colony occupy a two-dimensional region with one dimension being along the colony-agar interface (denoted by x) and the other dimension being the one perpendicular to the agar surface (denoted by z). Further, the components of forces experienced by cells along the excluded dimension are set to zero. Thus, the movement of cells is restricted to be within the x-z plane. This two-dimensional setting allows study of both radial and vertical colony expansion in a computationally tractable manner.

3.2 - Overall time iteration

We use our hybrid discrete-continuum model to simulate a colony growing on hard agar from time $t = 0$ to a final simulation time $t = T_{final}$ in hours. Such simulations are done through a time iteration with a uniform macro time step Δt and a total of $N_{overall}$ time steps, where $N_{overall}\Delta t = T_{final}$. Initially, we distribute glucose uniformly in the agar region with a constant glucose concentration $C_{g,0}$. To begin the simulation, we place one cell at the center of the agar surface. The initial velocity and angular velocity of this cell are set to be zero. In each time iteration, we simulate the colony growth for a time period Δt. Each such individual iteration with the time step Δt consists of the following main steps:

(1) Generate the macroscopic boundary of colony Γ_{01} (Fig. S16) by thresholding the cell density.
(2) Update the concentrations of glucose, oxygen, and acetate C_g, C_o, and C_a, respectively, by solving the system of reaction-diffusion equations for these concentrations using N_{conc} iterations with a small time step dt, where, $N_{conc}dt = \Delta t$.
(3) Update the local cell growth rate.
(4) Use the agent-based model to simulate the cell growth and division, compute the cell interaction forces and torques, and simulate the cell movement using the velocity-Verlet method (4), for all the cells in the colony with a small time step δt and a total number of iterations N_{cell} with $N_{cell}\delta t = \Delta t$.

We refer readers to Warren et al. (2019) (2) for further details on model aspects involving the agent-based treatment of growth, division, and movement of individual cells within the colony.

3.3 - Numerical solution to reaction-diffusion equations

Given the colony boundary Γ_{01} (Fig. S16) and concentrations C_g, C_o, and C_a of glucose, oxygen, and acetate, respectively, in both the agar region Ω_- and colony region Ω_+ at time $t = n\Delta t$ for some n $(0 \leq n < N_{overall})$, we solve the system of reaction-diffusion equations for the time period from $t = n\Delta t$ to $t = (n+1)\Delta t$ using N_{conc} iterations with each iteration having a time step dt to obtain all the concentrations at time $t = (n+1)\Delta t$. The initial values of the concentrations are those at the time $t = n\Delta t$ (i.e., the concentrations from the previous macro time step $n\Delta t$).

We use different methods of numerical discretization for solving the partial differential equations involved in the agar and colony regions. We use the Crank–Nicholson scheme for the time-dependent diffusion equation in the agar region Ω_- with the time step size dt, and use the forward Euler method to discretize the reaction-diffusion equation in the colony region Ω_+ with a smaller time step size dt/N_{colony} for some large integer N_{colony}. The two parts are coupled through the interface conditions, i.e., the continuity of the concentration and its flux across the agar-colony interface. We adopt the nested finite difference grids as in Warren et al. (2019) (2), with a fine grid covering the colony region and a region in agar beneath the colony, and several levels of coarser grids covering the rest of the agar region. For regular grid points inside the colony region (those grid points that are away from the colony surface), we use the standard five-point finite-difference scheme to discretize the diffusion operator. For those irregular points, the grid points inside the colony but are close to the colony surface, we introduce ghost grid points outside but close to the colony, and use interpolation to assign the values of concentration at these grid points, and then discretize the diffusion operator at irregular grid points inside the colony. In the agar region, we

discretize the diffusion operator using the standard five-point finite-difference scheme at the grid points that are not on the interface between the fine and coarse grids, and using the standard scheme and interpolation for the interface grid points.

For each time step, we use the multigrid method to solve the system of algebraic linear equations resulting from the time and spatial discretization in the agar region, and use the forward Euler method to find steady-state solutions to the reaction-diffusion equations in the colony region. Since we take the solution at the previous step as our initial condition, the number of forward Euler steps required for convergence is small.

Algorithm 1 Algorithm for numerically solving reaction-diffusion equations to capture metabolite dynamics during agent-based colony simulations

`Input` : Model and numerical parameters (see Supplementary Tables 2, 1), colony shape determined by the boundary Γ_{01} (Fig. S16), concentrations C_g, C_o ,and C_a from the previous macro time step.
`Output` : Concentrations C_g, C_o, and C_a in the agar and colony regions.
`Initialization:` Set $n = 0$.
while $n < N_{conc}$ **do**
- Use the multigrid method to solve the linear system of algebraic equations resulting from the time and spatial discretization of the reaction-diffusion equations in the agar region Ω_-.
- Update the concentrations C_g, C_o, and C_a at the colony-agar interface using the interface conditions.
- Use the forward Euler method to solve the reaction-diffusion equations for the steady state concentrations C_g, C_o, and C_a in the colony region Ω_+, with the time step dt/N_{colony};
- Update $n \leftarrow n + 1$.

end while

4 - Supplementary Note 4: Effects of (1+1)-dimensional geometry

To accommodate simulations of large colonies, both our agent-based model and the system of reaction-diffusion equations were restricted to (1+1)-dimensions. Below, we discuss the impact of such dimension reduction on the results of our simulations.

Dependence of solution to reaction-diffusion equations on model dimension

The reaction-diffusion component of our metabolic model allows us to obtain a comprehensive picture of the spatiotemporal dynamics of nutrient gradients as the simulated colony expands. To understand the effects of the reduced spatial dimension in our (1+1)-model on the dynamics of nutrient gradients (specifically glucose) in our simulations, we performed additional numerical studies using a simplified setting as described below.

In order to compare the glucose gradient predicted by a (1+1)-d model with a three dimensional, i.e. (2+1)-d model at a given time of colony development, we construct approximate (1+1) and (2+1)-dimensional colony shapes (triangular and conical respectively) based on experimental colony dimensions. Based on the constructed shape corresponding to the given time, the agar region (Ω_-) and colony region (Ω_+) of the computational domain is determined for each geometry. Then the steady-state solution to the reaction-diffusion equations for glucose concentration (described below) with both (1+1) and (2+1) dimensional geometry are solved. The local glucose concentration is denoted by $C_g := C_g(x, z)$ and $C_g := C_g(r, z)$ for (1+1) and (2+1)-dimensional models respectively and we solve for the steady-state solution of the following equations which represent a simplified form of equations [5]–[7].

$$D_{g,-}\Delta C_g = 0 \qquad \text{in agar region } \Omega_-, \qquad [34]$$

$$D_{g,+}\Delta C_g = \rho q_g \lambda_g \frac{C_g}{C_g + K_g} \qquad \text{in colony region } \Omega_+. \qquad [35]$$

$$C_{g,-} = C_{g,+} \qquad \text{on colony-agar interface } \Gamma_{12}, \qquad [36]$$

$$D_{g,-}\partial_z C_{g,-} = D_{g,+}\partial_z C_{g,+} \qquad \text{on colony-agar interface } \Gamma_{12}, \qquad [37]$$

$$C_g = C_{g,0} \qquad \text{on } \Gamma_s \qquad [38]$$

$$\partial_n C_g = 0 \qquad \text{on } \Gamma_b \cup \Gamma_{02} \cup \Gamma_{01}. \qquad [39]$$

Here, $D_{g,-}$ and $D_{g,+}$ are the diffusion coefficients for glucose in agar and colony, respectively, ρ is the local cell density within the colony which is assumed to be a constant. $q_{g,ana}$ and $\lambda_{g,ana}$ are the specific glucose uptake flux and the maximum growth rate, respectively. Since anaerobic glucose consumption is dominant within colony, for simplicity, we take $q = q_{g,ana}$ and $\lambda = \lambda_{g,ana}$. K_g represents the Monod constant for the glucose, and ∂_n denotes the normal derivative. The values of these parameters are listed in Supplementary Table 1. Note that the Laplacian operator, Δ differs between the $(1+1)$ and $(2+1)$-geometries, i.e.,

$$\Delta C_g = \partial_{xx} C_g + \partial_{zz} C_g \qquad \text{for } (1+1)\text{-geometry;}$$

$$\Delta C_g = \partial_{rr} C_g + \partial_{zz} C_g + \frac{1}{r}\partial_r C_g \qquad \text{for } (2+1)\text{-geometry (cylindrical symmertry is assumed).}$$

Further, the concentration $C_{g,0}$ on the far boundaries of the agar region (Γ_s), i.e., the glucose concentration far away from the colony is taken to be constant.

Numerical methods for steady-state solution

We use finite differences to discretize the reaction-diffusion equations and boundary conditions (equations [34]-[39]). For an interior grid point, such discretization is done by using central differencing. For a grid point near the colony-air interface but is outside the colony region, we assign a value of glucose concentration by interpolating the values of such concentration at nearby grid points inside the colony. Upon applying the finite difference scheme, the equations are discretized into a system of algebraic equations which can be represented as,

$$F(\mathbf{c}) := A\mathbf{c} + \mathbf{b} - f(\mathbf{c}) = 0.$$

where, **c** is a vector representing the values of concentration C_g at all the grid points, A is the coefficient matrix for the linear part of the discretized equations, **b** is a constant vector that corresponds to the constant terms in the discretized equations and $f(\mathbf{c})$ is the nonlinear term resulting from the discretization. We solve this nonlinear system of algebraic equations using Newton's iteration, i.e.,

$$\mathbf{c}^{(n+1)} = \mathbf{c}^{(n)} - [\nabla F(\mathbf{c}^{(n)})]^{-1} F(\mathbf{c}^{(n)}), \quad n = 0, 1, \ldots,$$

with the initial guess being $\mathbf{c}^{(0)} = \mathbf{0}$. Note that $\nabla F(\mathbf{c}^{n}) = A - \nabla f(\mathbf{c}^{n})$. Thus, equivalently, given $\mathbf{c}^{(n)}$, we solve the following linear system of equations to get $\mathbf{c}^{(n+1)}$:

$$\nabla F(\mathbf{c}^{(n)})\mathbf{c}^{(n+1)} = [\nabla F(\mathbf{c}^{(n)})]\mathbf{c}^{(n)} - F(\mathbf{c}^{(n)}). \tag{40}$$

Algorithm 2 Algorithm for obtaining steady state solution for the reaction-diffusion system to study effects of dimension reduction

```
Input : Model and numerical parameters, the colony shape set by Γ_01, and a tolerance ε.
Output : Vector c that represents the concentrations C_g in the agar and colony regions.
Initialization: Set c^(0) = 0 and n = 0.
while ||c^(n) − c^(n−1)|| ≥ ε do
    • Calculate matrix ∇f(c^(n)) and vector F(c^(n)).
    • Solve [40] for c^(n+1).
    • Update n ← n + 1.
end while
```

Effects of dimension on glucose gradient

Our findings based on simulations of the model described above indicate quantitative differences in nutrient concentration profiles between (1+1)-d simulations and (2+1)-d simulations (Fig. S18, S19). However, irrespective of the dimensions in which the reaction-diffusion system was solved, the glucose concentration in the agar-colony interface is saturating i.e., $\sim$ an order of magnitude higher than the Monod constant for both 10 mM and 20 mM boundary condition for glucose concentration till 60 h (Fig. S18). Further, irrespective of the dimensionality, glucose concentration drops sharply below the Monod constant with an increasing vertical distance into the colony (Fig. S19). Moreover, the vertical penetration of glucose into the colony is higher for 20 mM glucose condition than 10 mM glucose condition for both geometries (Fig. S19gh, even though the (2+1)-d solution has quantitatively a higher glucose penetration overall than the (1+1)-d solution (Fig. S19gh). Thus, our findings based on the agent-based hybrid simulations that radial colony expansion is not expected to be nutrient limited, while colony vertical expansion being limited by glucose penetration remains true irrespective of the dimensions in which the reaction-diffusion equations are solved in.

Limitations of (1+1)-dimensional geometry of agent based model

In our simulations the orientation of individual cells is forced to be within the x-z plane. Thus, potential effects of cell orientation along the third dimension (y direction) will not be captured by our (1+1)-dimensional agent based model. For instance, the cells in the monolayer of our simulations are forced to point their long axis radially outwards (Fig. 3a in main text) unlike in experiments (Fig. 3kl in main text, Fig. S5 and Supplementary Movies 3,4) which lead to an overestimation of the monolayer width in our simulations (Fig. 3m - experiments and Fig. 3g - simulations). Further, in experimental colonies, the verticalized cells in colony interior could potentially have a tilt along the y-direction which is excluded in our (1+1)-dimensional simulations. Any minor tilt of cells along the y-axis would be expected to contribute to more growth and colony expansion along the y-direction. This would result in a more rounded colony shape like observed in experiments (Fig. 1a,b,c in main text) as opposed to the very triangular shaped colonies arising in our agent-based simulations restricted to a (1+1)-dimensional geometry (Fig. 2g,h,i in main text).

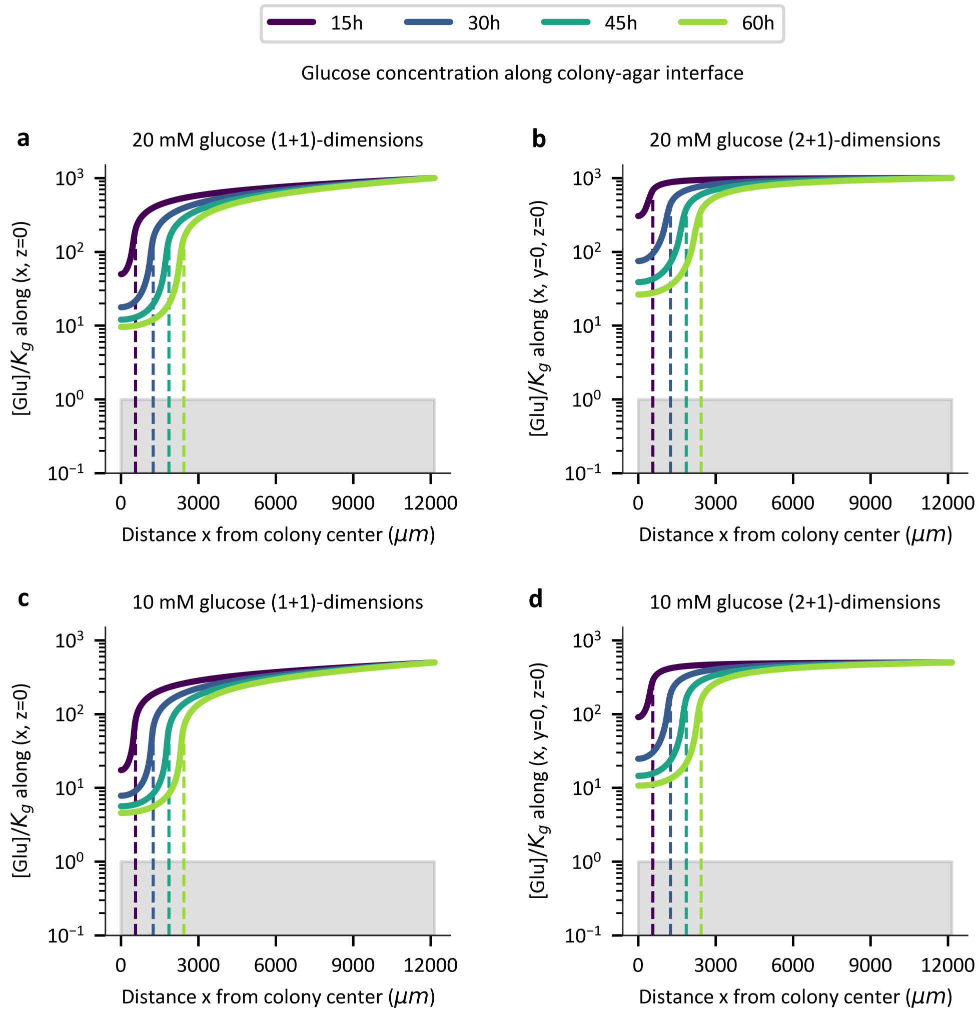

Fig. S18: Glucose concentration along colony-agar interface (z=0) for **(a,b)** 20 mM glucose boundary condition and **(c,d)** 10 mM glucose boundary condition at various times (coded by color) of colony development obtained for **(a,c)** (1+1)-geometry and **(b,d)** (2+1) geometry.

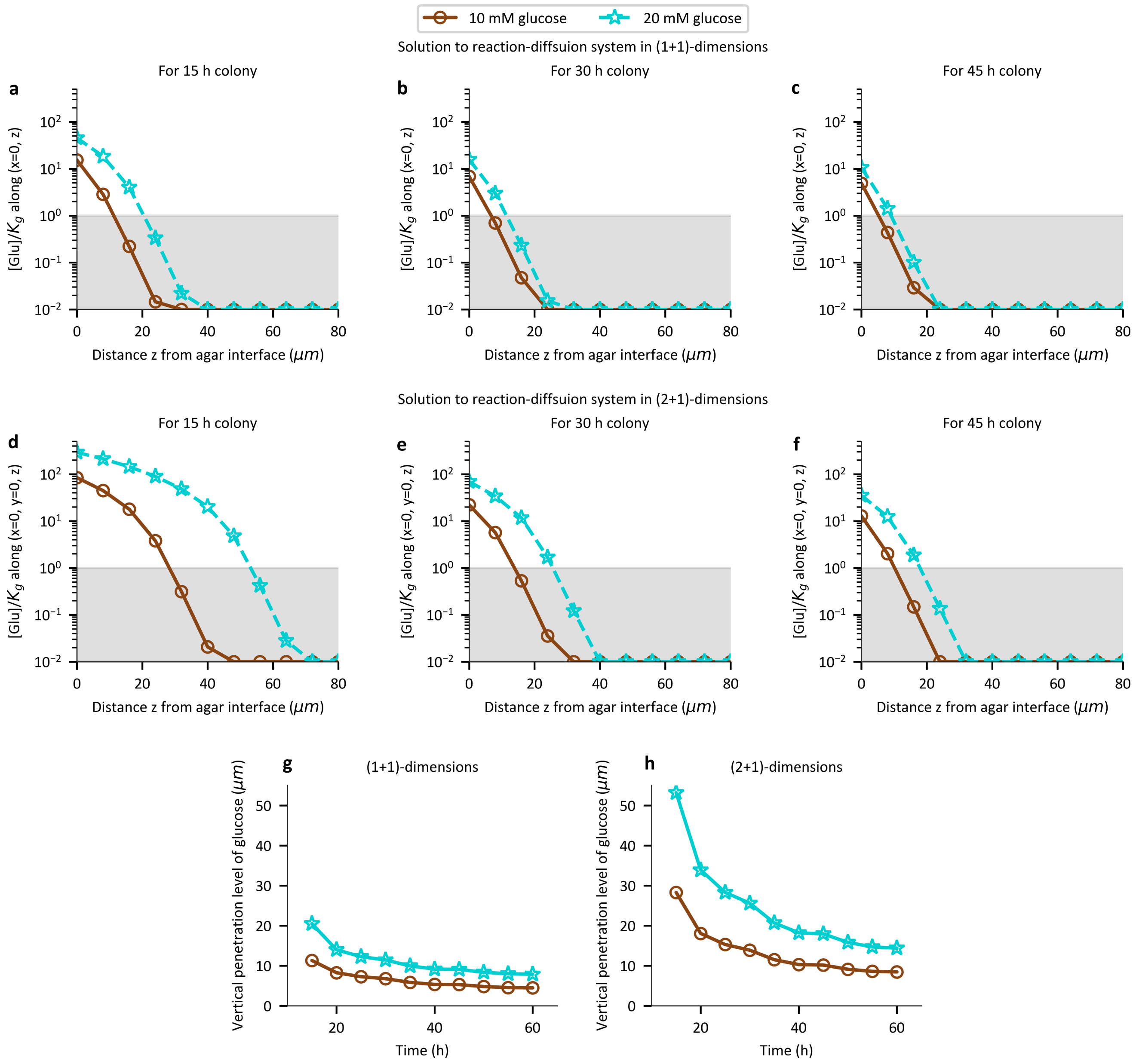

Fig. S19: Glucose concentration along vertical axis (0,z) for 10 mM and 20 mM glucose boundary conditions using (1+1)-geometry at **(a)** 15 h, **(b)** 30 h and **(c)** 45 h of colony development. Glucose concentration along vertical axis (0,0, z) for 10 mM and 20 mM glucose boundary conditions using (2+1)-geometry at **(d)** 15 h, **(e)** 30 h and **(f)** 45 h of colony development. The vertical penetration level of glucose, i.e., the height z inside the colony (measured from the colony center) at which glucose concentration drops below the Monod constant value K_g for 10 mM and 20 mM glucose boundary conditions using **(g)** (1+1)-geometry and **(h)** (2+1) geometry at various times.

5 - Supplementary Note 5: The effect of buffer concentration on toxicity due to acetate excretion

Cells excrete acetate during fermentation and overflow metabolism. This excreted acetate can potentially lower pH, and also inhibit cell growth (5; 8). In Ref. (5; 8), it was found the toxic effect of acetate on cell growth depends quantitatively on the concentration of acetic acid, and an empirical relationship between cell growth rate and [HAc] was obtained as,

$$\lambda([\mathrm{HAc}]) = \sqrt{\frac{[\mathrm{HAc}]}{4.89\ \mathrm{mM}}} \qquad [41]$$

where [HAc] is expressed in units of mM. Thus, to determine the magnitude of toxicity to cell growth due to acetate in a buffer solution of known concentration, the concentration of acetate in the form of acetic acid [HAc] needs to be determined. For simplicity, we consider the dissociation equations of water (H_2O), acetic acid (HAc) and a buffer which is a weak acid (represented by HB). Note that the weak acid buffer here represents a simplification of potassium based phosphate buffer which is used in our experiments (see Methods in main text). A phosphate buffer has three disassociation constants since phosphoric acid is triprotic. However, in our experiments the buffer is prepared at pH $\sim$7, and so the relevant pKa is 6.8. Thus in our equations we take the corresponding dissociation constant K_b as $10^{-6.8}$ M. The chemical equations involved are,

$$H_2O \longrightarrow H^+ + OH^- \qquad [42]$$

$$HAc \longrightarrow H^+ + Ac^- \qquad [43]$$

$$HB \longrightarrow H^+ + B^- \qquad [44]$$

The corresponding equilibrium and conservation equations involved can be written down as,

$$\frac{K_w}{c_1} = \frac{[OH^-][H^+]}{[H_2O]} \quad \text{where, } K_w{=}10^{-14}\,\mathrm{M}^2 \text{ and } [H_2O] \approx c_1 = 55\,\mathrm{M} \qquad [45]$$

$$K_{ac} = \frac{[Ac^-][H^+]}{[HAc]} \quad \text{where, } K_{ac}{=}10^{-4.75}\,\mathrm{M} \qquad [46]$$

$$K_b = \frac{[B^-][H^+]}{[HB]} \qquad [47]$$

$$c_1 = [H_2O] + [OH^-] \quad \text{where } c_1{=}55\,\mathrm{M} \qquad [48]$$

$$C_{ac} = [HAc] + [Ac^-] \qquad [49]$$

$$C_b = [HB] + [B^-] \qquad [50]$$

$$[H^+] + \alpha = [OH^-] + [Ac^-] + [B^-] \qquad [51]$$

Here, C_{ac} and C_b respectively represent the total concentration of acetate and the buffer B respectively. K_w, K_{ac} and K_b represent the equilibrium constants of water, acetic acid and the weak acid buffer. Further, α represents the charge contribution of other existing species apart from $[H^+]$, $[OH^-]$, and $[B^-]$ in the buffer solution. In our experiments, we prepare the phosphate buffer solution such that the pH is neutral (see Methods in main text). Thus, for a buffer solution at a given concentration C_b, the pH is 7 before the addition of acetate, which implies $[H^+] = [OH^-] = 10^{-7}$,$[Ac^-] = 0$, and $[B^-]$ satisfies $\frac{[H^+][B^-]}{C_b - [B^-]}{=}K_b$. So,

$$\alpha{=} -[H^+] + [OH^-] + [Ac^-] + [B^-] = [B^-] = \frac{C_b K_b}{[H^+] + K_b}$$

Now, upon algebraic simplification of the above conservation and equilibrium equations, we get the following system of equations.

$$[OH^-]\left([OH^-] + [Ac^-] + [B^-] - \frac{C_b K_b}{[H^+] + K_b}\right) = \frac{K_w}{c_1}\left(c_1 - [OH^-]\right) \qquad [52]$$

$$[Ac^-]\left([OH^-] + [Ac^-] + [B^-] - \frac{C_b K_b}{[H^+] + K_b}\right) = K_{ac}\left(C_{ac} - [Ac^-]\right) \qquad [53]$$

$$[B^-]\left([OH^-] + [Ac^-] + [B^-] - \frac{C_b K_b}{[H^+] + K_b}\right) = K_b\left(C_b - [B^-]\right) \qquad [54]$$

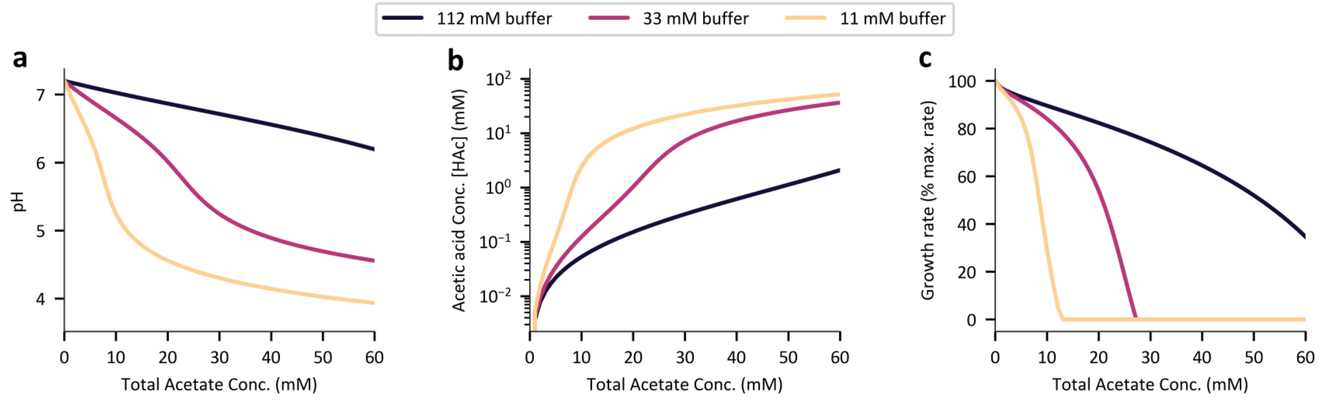

Fig. S20: (a) The pH, **(b)** acetic acid concentration and **(c)** growth rate (given by equation 41) is plotted against the total acetate concentration for different buffer concentrations (indicated by color). These values are obtained by solving the system of equations (Eq.52-54) at various total acetate concentrations (C_{ac}) between 0 mM and 60 mM.

The above three equations form a closed system of quadratic equations with unknowns $[\text{OH}^-], [\text{Ac}^-], [\text{B}^-]$. Thus, given the total buffer concentration C_b and total acetate concentration C_{ac}, the concentration $[\text{Ac}^-]$ can be solved numerically using Newton's iterative algorithm . Then, once $[\text{Ac}^-]$ is obtained the acetic acid concentration is found as $[\text{HAc}] = \text{C}_{\text{ac}} - [\text{Ac}^-]$.

Relationship between buffer concentration and acetate toxicity

In our experiments, the initial pH of the medium is set to 7.2 with a phosphate buffer at a very high concentration of 112 mM. Excretion of short chain fatty acids (primarily acetate) by cells metabolizing glucose within the colony could potentially cause acid stress. Since the only external carbon source provided is glucose, at 20 mM glucose, an upper bound for acetate concentration is $\sim$45 mM (based on anaerobic glucose metabolism $\sim$2.3 mM of SCFA is excreted per 1 mM of glucose consumed (5; 8). Note that 45mM is a gross over-estimate for acetate concentration since glucose consumption within the colony is a combination of aerobic and anaerobic metabolism and acetate excretion is much lower during aerobic glucose metabolism. Indeed, based on our simulations, the local acetate concentration level inside the colony is expected to be at maximum $\sim$8 mM (Fig. 5 in main text). At these low acetate levels, the pH is expected to remain near-neutral, and the growth rate of cells is expected to be unaffected for a buffer concentration of either 112 mM or even lower at 33 mM (Fig. S20).

Supplementary Tables

The parameters involved in the metabolic model are listed below in Supplemetary Table 1. Numerical parameters involved in solving the partial differential reaction-diffusion equations for nutrient concentrations are listed separately in Supplementary Table 2. The model and numerical parameters for the agent-based discrete simulations for cellular mechanical interaction forces are similar to values used in Warren et al. (2019) (2).

Supplementary Table 1

Table 1: Parameters for metabolic and reaction-diffusion model.

Symbol	Description	Value	Units	Source (or) Rationale for choice
$\lambda_{g,aer}$	Maximum batch culture growth rate aerobically on glucose	0.9	h^{-1}	This study
$\lambda_{g,ana}$	Maximum batch culture growth rate anaerobically on glucose	0.6	h^{-1}	This study
$\lambda_{a,aer}$	Maximum batch culture growth rate aerobically on acetate	0.4	h^{-1}	This study
$q_{g,aer}$	Specific flux for glucose during aerobic growth on glucose	11	$mmol/g_{DW}$	(5)
$q_{g,ana}$	Specific flux for glucose during anaerobic growth on glucose	28	$mmol/g_{DW}$	(5)
$q_{a,aer}$	Specific flux for acetate during aerobic growth on acetate	33	$mmol/g_{DW}$	Equiv. to $q_{g,aer}$ on a per C-atom basis
$q_{o,g}$	Specific flux for oxygen during aerobic growth on glucose	22	$mmol/g_{DW}$	(6)
$q_{o,a}$	Specific flux for oxygen during aerobic growth on acetate	22	$mmol/g_{DW}$	(6)
$p_{a,aer}$	Specific flux of acetate excretion during aerobic growth on glucose	3	$mmol/g_{DW}$	(7)
$p_{a,ana}$	Specific flux of acetate excretion during anaerobic growth on glucose	16	$mmol/g_{DW}$	(8)
$q^0_{g,aer}$	Maintenance rate for glucose in aerobic condition	1	$mmol/g_{DW}/h$	(5)
$q^0_{g,ana}$	Maintenance rate for glucose in anaerobic condition	10	$mmol/g_{DW}/h$	(5)
$q^0_{a,aer}$	Maintenance rate for acetate in aerobic condition	3	$mmol/g_{DW}/h$	Equiv. to $q^0_{g,aer}$ on a per C-atom basis
$q^0_{o,g}$	Uptake rate for oxygen during maintenance on glucose in aerobic condition	2	$mmol/g_{DW}/h$	Approximated as $q_{o,g} \cdot \frac{q^0_{g,aer}}{q_{g,aer}}$
$q^0_{o,a}$	Uptake rate for oxygen during maintenance on acetate in aerobic condition	2	$mmol/g_{DW}/h$	Approximated as $q_{o,a} \cdot \frac{q^0_{a,aer}}{q_{a,aer}}$
$p^0_{a,ana}$	Acetate excretion rate during anaerobic maintenance on glucose	10	$mmol/g_{DW}/h$	$\approx q^0_{g,ana}$ (Ref. (9) 1 Glu → 1 Acetate + 1 Ethanol + 2 Formate)
K_g	Monod constant for glucose	20	μM	(10)
K_a	Monod constant for acetate	5	mM	This study (see Fig. 5e in main text)
K_o	Monod constant for oxygen	0.1	μM	(11)
$C_{o,0}$	Boundary value of oxygen concentration	260	μM	(11)
$D_{g,-}$	Diffusion coefficient for glucose in agar	740	$\mu m^2/s$	(11)
$D_{g,+}$	Diffusion coefficient for glucose in colony	110	$\mu m^2/s$	(2) : $D_{g,+} \approx \phi D_{g,-}$ with $\phi \approx 0.15$
$D_{a,-}$	Diffusion coefficient for acetate in agar	1100	$\mu m^2/s$	(11)
$D_{a,+}$	Diffusion coefficient for acetate in colony	165	$\mu m^2/s$	(2) : $D_{a,+} \approx \phi D_{a,-}$ with $\phi \approx 0.15$
$D_{o,-}$	Diffusion coefficient for oxygen in agar	2500	$\mu m^2/s$	(11)
$D_{o,+}$	Diffusion coefficient for oxygen in colony	375	$\mu m^2/s$	(2) : $D_{o,+} \approx \phi D_{o,-}$ with $\phi \approx 0.15$

Supplementary Table 2

Table 2: Numerical parameters and cell-centric parameters.

Symbol	Description	Value	unit
L	Half-Length of agar region (Fig. S16)	12160	μm
a	Depth of agar region (Fig. S16)	8192	μm
b	Height of air region (Fig. S16)	400	μm
N_x	Number of grid points in the x direction	1520	No unit
$N_{z\text{c}}$	Number of grid points in the z direction in colony	100	No unit
$N_{z\text{a}}$	Number of grid points in the z direction in agar	512	No unit
$h_{\text{grid,c}}$	Spatial grid size in colony region	4	μm
M_{agar}	Number of grid levels in agar region (Multi-nested grid approach (2))	3	No unit
Δt	Macro-time step for simulations (see Supp. Note 3.2)	0.01	h
dt	Time step while solving reaction-diffusion equations (see Supp. Note 3.2)	0.00004	h
δt	Time step to simulate cell growth and movement due to forces (see Supp. Note 3.2)	0.00001	h
N_{colony}	Maximum number of iterations for forward-Euler method to solve PDEs in colony region (see Supp. Note 3.3)	10000	No unit
w_0	Diameter of hemispherical caps of a cell	1	μm
ΔL	Length added to a cell before division occurs	2	μm
l_{ran}	Max. length fluctuation after cell division	0.125	μm
ϕ_{ran}	Max. angle fluctuation after cell division	0.0005	$radians$
ω_{ran}	Max. angular velocity fluctuation after cell division	0.0005	$radians.h^{-1}$
ρ_{cell}	Cell dry weight per cell volume	0.25	$pg/\mu m^3$

Supplementary Table 3

Table 3: Parameters for force calculations. The agent-based model for cell-mechanics are inherited from Warren et al (2). Very little is experimentally known about the parameters involved here in the context of *E. coli* colony growth and the definition of these parameters are described in Warren et al. For our study, the values of these parameters were chosen to be similar to the values used in Warren et al with minor modifications such that the colony dimensions (radius and height) in our (1+1)-dimensional setting are roughly comparable in magnitude to experimental colonies at similar times.

Symbol	Description	Value	unit
μ_{cc}	Cell-cell friction coefficient	0.1	no unit
μ_{ca}	Cell-agar friction coefficient	0.4	no unit
k_{cc}	Cell-cell Hertzian elastic coefficient	50000	$pg.\mu m^{-1}.h^2$
k_{ca}	Cell-agar Hertzian elastic coefficient	50000	$pg.\mu m^{-1}.h^2$
$\gamma_{cc,n}$	Cell-cell normal dissipation rate	100	$\mu m^{-1}.h^{-1}$
$\gamma_{cc,t}$	Cell-cell tangential dissipation rate	10000	$\mu m^{-1/2}.h^{-1}$
$\gamma_{ca,n}$	Cell-agar normal dissipation rate	100	$\mu m^{-1}.h^{-1}$
$\gamma_{ca,t}$	Cell-agar tangential dissipation rate	10000	$\mu m^{-1/2}.h^{-1}$
γ_{surf}	Surface tension constant	125	$pg.h^{-2}$
δh	Tightness of water sticking to cells	-0.01	μm
μ_{liq}	Liquid viscosity	0.025	$pg.\mu m^{-1}.h^{-1}$
h_{ran}	Maximum height fluctuation of agar surface	0.1	μm

Supplementary References

[1] P. E. Schavemaker and M. Lynch, "Flagellar energy costs across the tree of life," *elife*, vol. 11, p. e77266, 2022.

[2] M. Warren, H. Sun, Y. Yan, J. Cremer, B. Li, and T. Hwa, "Spatiotemporal establishment of dense bacterial colonies growing on hard agar," *eLife*, vol. 8, p. e41093, 2019.

[3] S. Taheri-Araghi, S. Bradde, J. T. Sauls, N. S. Hill, P. A. Levin, J. Paulsson, M. Vergassola, and S. Jun, "Cell-size control and homeostasis in bacteria," *Current biology*, vol. 25, no. 3, pp. 385–391, 2015.

[4] D. Frenkel and B. Smit, *Understanding Molecular Simulation*. Academic Press, 2002.

[5] B. R. Taylor, *Physiology of bacteria in anaerobic environments*. PhD thesis, UC San Diego, 2022.

[6] K. B. Andersen and K. von Meyenburg, "Are growth rates of Escherichia coli in batch cultures limited by respiration?," *Journal of bacteriology*, vol. 144, no. 1, pp. 114–123, 1980.

[7] M. Basan, S. Hui, H. Okano, Z. Zhang, Y. Shen, J. R. Williamson, and T. Hwa, "Overflow metabolism in Escherichia coli results from efficient proteome allocation," *Nature*, vol. 528, no. 7580, pp. 99–104, 2015.

[8] B. R. Taylor, V. Patsalo, H. Okano, Y. Shen, Z. Zhange, J. R. Williamson, J. D. Rabinowitz, and T. Hwa, "A metabolic sum rule dictates bacterial response to short-chain fatty acid stress," *bioRxiv*, pp. 2022–08, 2022.

[9] M. Mori, C. Cheng, B. R. Taylor, H. Okano, and T. Hwa, "Functional decomposition of metabolism allows a system-level quantification of fluxes and protein allocation towards specific metabolic functions," *Nature Communications*, vol. 14, no. 1, p. 4161, 2023.

[10] J. Monod, "The growth of bacterial cultures," *Annual review of microbiology*, vol. 3, no. 1, pp. 371–394, 1949.

[11] J. A. Cole, L. Kohler, J. Hedhli, and Z. Luthey-Schulten, "Spatially-resolved metabolic cooperativity within dense bacterial colonies," *BMC systems biology*, vol. 9, no. 1, pp. 1–17, 2015.
