# Supplementary material for: Spatiotemporal development of expanding bacterial colonies driven by emergent mechanical constraints and nutrient gradients
Source: Nat Commun. 2025 May 26;16:4878. doi: 10.1038/s41467-025-60004-z (PMC12106844; doi:10.1038/s41467-025-60004-z)
Supplement: Supplementary file 2 — Description of Additional Supplementary Files [file 41467_2025_60004_MOESM2_ESM.pdf]

Description of Additional Supplementary Files:

Supplementary Movie 1: 3D rendering from confocal microscopy images of a ~20 h old EQ59 colony grown on a 1.5 % (w/v) minimal media agar plate prepared with 20 mM glucose, 10 mM ammonium chloride, and 112 mM phosphate buffer.

Supplementary Movie 2: 3D rendering from confocal microscopy images of a ~53 h old EQ59 colony grown on a 1.5 % (w/v) minimal media agar plate prepared with 20 mM glucose, 10 mM ammonium chloride, and 112 mM phosphate buffer.

Supplementary Movie 3: z-stack images of GFP fluorescence in the peripheral region of a ~2-day old EQ59 colony grown on a 1.5 % (w/v) minimal media agar plate prepared with 20 mM glucose, 10 mM ammonium chloride, and 112 mM phosphate buffer. The z-coordinate corresponding to each z-frame is shown in top right corner.

Supplementary Movie 4: Brightfield z-stack images of peripheral region of a ~2-day old EQ59 colony grown on a 1.5 % (w/v) minimal media agar plate prepared with 20 mM glucose, 10 mM ammonium chloride, and 112 mM phosphate buffer. The z-coordinate corresponding to each z-frame is shown in top right corner.

Supplementary Movie 5: Spatiotemporal dynamics of glucose concentration (in units of K_g) during simulated colony development in the colony region and the rectangular sub-portion of agar region immediately beneath colony.

Supplementary Movie 6: Spatiotemporal dynamics of oxygen concentration (in units of K_o) during simulated colony development in the colony region and the rectangular sub-portion of agar region immediately beneath colony.

Supplementary Movie 7: Spatiotemporal dynamics of acetate concentration (in units of K_a) during simulated colony development in the colony region and the rectangular sub-portion of agar region immediately beneath colony.

Supplementary Movie 8: Spatiotemporal dynamics of cell growth rate (h^{-1}) during simulated colony development.

Supplementary Movie 9: Spatiotemporal dynamics of cell growth rate (h^{-1}) from (Top) aerobic glucose metabolism, (Middle) anaerobic glucose metabolism, (Bottom) aerobic acetate metabolism during simulated colony development.

Supplementary Movie 10: Spatiotemporal dynamics of maintenance flux (mmol/gdw/h) from (Top) glucose metabolism, (Bottom) acetate metabolism during simulated colony development.

Supplementary Movie 11: Spatiotemporal dynamics of carbon starvation within colony during simulated colony development.

Supplementary Movie 12: Spatiotemporal dynamics of the death zone within colony during simulated colony development.
