# Supplementary material for: Spatiotemporal development of expanding bacterial colonies driven by emergent mechanical constraints and nutrient gradients
Source: Nat Commun. 2025 May 26;16:4878. doi: 10.1038/s41467-025-60004-z (PMC12106844; doi:10.1038/s41467-025-60004-z)
Supplement: Supplementary file 15 — Reporting Summary [file 41467_2025_60004_MOESM15_ESM.pdf]

nature portfolio

Corresponding author(s): Dr. Terence Hwa

Last updated by author(s): Apr 13, 2024

Reporting Summary

Nature Portfolio wishes to improve the reproducibility of the work that we publish. This form provides structure for consistency and transparency in reporting. For further information on Nature Portfolio policies, see our Editorial Policies and the Editorial Policy Checklist.

Statistics

For all statistical analyses, confirm that the following items are present in the figure legend, table legend, main text, or Methods section.

n/a	Confirmed	
☐	☒	The exact sample size (n) for each experimental group/condition, given as a discrete number and unit of measurement
☐	☒	A statement on whether measurements were taken from distinct samples or whether the same sample was measured repeatedly
☒	☐	The statistical test(s) used AND whether they are one- or two-sided<br>*Only common tests should be described solely by name; describe more complex techniques in the Methods section.*
☒	☐	A description of all covariates tested
☒	☐	A description of any assumptions or corrections, such as tests of normality and adjustment for multiple comparisons
☒	☐	A full description of the statistical parameters including central tendency (e.g. means) or other basic estimates (e.g. regression coefficient) AND variation (e.g. standard deviation) or associated estimates of uncertainty (e.g. confidence intervals)
☒	☐	For null hypothesis testing, the test statistic (e.g. F, t, r) with confidence intervals, effect sizes, degrees of freedom and P value noted<br>*Give P values as exact values whenever suitable.*
☒	☐	For Bayesian analysis, information on the choice of priors and Markov chain Monte Carlo settings
☒	☐	For hierarchical and complex designs, identification of the appropriate level for tests and full reporting of outcomes
☒	☐	Estimates of effect sizes (e.g. Cohen's d, Pearson's r), indicating how they were calculated

Our web collection on statistics for biologists contains articles on many of the points above.

Software and code

Policy information about availability of computer code

Data collection	Leica LAS AF 4.0.0.11706 software for collection of confocal microscopy data, custom MATLAB 2017b
Data analysis	C++11, openMP, python 3.8.19, numpy 1.24.3, scipy 1.10.1, pandas 2.0.3, matplotlib 3.7.2, opencv-python 4.9.0.80, ImageJ Version 1.54p (Fiji), MATLAB R2021b

For manuscripts utilizing custom algorithms or software that are central to the research but not yet described in published literature, software must be made available to editors and reviewers. We strongly encourage code deposition in a community repository (e.g. GitHub). See the Nature Portfolio guidelines for submitting code & software for further information.

Data

Policy information about availability of data

All manuscripts must include a data availability statement. This statement should provide the following information, where applicable:

- Accession codes, unique identifiers, or web links for publicly available datasets
- A description of any restrictions on data availability
- For clinical datasets or third party data, please ensure that the statement adheres to our policy

Quantitative data underlying graphs in the figures are provided as source data with this paper. Raw microscopy images which were analyzed to generate quantitative data are available upon request from the corresponding author.

Research involving human participants, their data, or biological material

Policy information about studies with human participants or human data. See also policy information about sex, gender (identity/presentation), and sexual orientation and race, ethnicity and racism.

Reporting on sex and gender	n/a
Reporting on race, ethnicity, or other socially relevant groupings	n/a
Population characteristics	n/a
Recruitment	n/a
Ethics oversight	n/a

Note that full information on the approval of the study protocol must also be provided in the manuscript.

Field-specific reporting

Please select the one below that is the best fit for your research. If you are not sure, read the appropriate sections before making your selection.

[x] Life sciences [ ] Behavioural & social sciences [ ] Ecological, evolutionary & environmental sciences

For a reference copy of the document with all sections, see nature.com/documents/nr-reporting-summary-flat.pdf

Life sciences study design

All studies must disclose on these points even when the disclosure is negative.

Sample size	n>=3 independent colonies growing on different agar plates were used for tracking colony growth dynamics in each condition. Colony growth has been observed to robust and reproducible by several other studies in the past. Thus, n>=3 replicates was deemed to be the appropriate sample size by us.
Data exclusions	Results with 40 mM initial glucose concentration was excluded in the manuscript due to technical difficulties with simulations for 40 mM glucose concentration. Since a high glucose concentration leads to a higher number of cells in the colony, the simulation required a large computational demand and made simulating colony growth beyond 40 h extremely slow.
Replication	All colony dynamics experiments central to the findings of this study were confirmed to be reproducible with n>=3 independent replicates. Colony growth has been observed to robust and reproducible by several other studies in the past.
Randomization	This is not applicable to us as our study concerns characterizing the development of monoclonal bacterial colonies. The phenomenological nature of experiments do not permit for randomization.
Blinding	This is not applicable to us as our study concerns characterizing the development of monoclonal bacterial colonies. The phenomenological nature of experiments do not permit for blinding.

Reporting for specific materials, systems and methods

We require information from authors about some types of materials, experimental systems and methods used in many studies. Here, indicate whether each material, system or method listed is relevant to your study. If you are not sure if a list item applies to your research, read the appropriate section before selecting a response.

Materials & experimental systems

n/a	Involved in the study	
[x]	[ ]	Antibodies
[x]	[ ]	Eukaryotic cell lines
[x]	[ ]	Palaeontology and archaeology
[x]	[ ]	Animals and other organisms
[x]	[ ]	Clinical data
[x]	[ ]	Dual use research of concern
[x]	[ ]	Plants

Methods

n/a	Involved in the study	
[x]	[ ]	ChIP-seq
[x]	[ ]	Flow cytometry
[x]	[ ]	MRI-based neuroimaging

Plants

Seed stocks	n/a
Novel plant genotypes	n/a
Authentication	n/a
